# Discrimination between 34 of 36 Possible Combinations of Three C>T SNP Genotypes in the *MGMT* Promoter by High Resolution Melting Analysis Coupled with Pyrosequencing Using A Single Primer Set

**DOI:** 10.3390/ijms222212527

**Published:** 2021-11-20

**Authors:** Katja Zappe, Christine Pirker, Heidi Miedl, Martin Schreiber, Petra Heffeter, Georg Pfeiler, Stefan Hacker, Werner Haslik, Sabine Spiegl-Kreinecker, Margit Cichna-Markl

**Affiliations:** 1 Department of Analytical Chemistry, Faculty of Chemistry, University of Vienna, 1090 Vienna, Austria; katja.zappe@univie.ac.at; 2 Department of Medicine I, Institute of Cancer Research, Medical University of Vienna, 1090 Vienna, Austria; christine.pirker@meduniwien.ac.at (C.P.); petra.heffeter@meduniwien.ac.at (P.H.); 3 Comprehensive Cancer Center, Medical University of Vienna, 1090 Vienna, Austria; heidi.miedl@meduniwien.ac.at (H.M.); martin.schreiber@meduniwien.ac.at (M.S.); 4 Department of Obstetrics and Gynecology, Medical University of Vienna, 1090 Vienna, Austria; 5 Department of Obstetrics and Gynecology, Division of Gynecology and Gynecological Oncology, Medical University of Vienna, 1090 Vienna, Austria; georg.pfeiler@meduniwien.ac.at (G.P.); werner.haslik@meduniwien.ac.at (W.H.); 6 Department of Plastic and Reconstructive Surgery, Medical University of Vienna, 1090 Vienna, Austria; stefan.hacker@gmail.com; 7 Department of Plastic, Reconstructive and Aesthetic Surgery, Landesklinikum Wiener Neustadt, 2700 Wiener Neustadt, Austria; 8 Department of Neurosurgery, Medical Faculty, Kepler University Hospital GmbH, Johannes Kepler University Linz, 4040 Linz, Austria; sabine.spiegl-kreinecker@kepleruniklinikum.at

**Keywords:** genotyping, multiple SNPs, *MGMT*, high resolution melting, pyrosequencing

## Abstract

Due to its cost-efficiency, high resolution melting (HRM) analysis plays an important role in genotyping of candidate single nucleotide polymorphisms (SNPs). Studies indicate that HRM analysis is not only suitable for genotyping individual SNPs, but also allows genotyping of multiple SNPs in one and the same amplicon, although with limited discrimination power. By targeting the three C>T SNPs rs527559815, rs547832288, and rs16906252, located in the promoter of the O6-methylguanine-DNA methyltransferase (*MGMT*) gene within a distance of 45 bp, we investigated whether the discrimination power can be increased by coupling HRM analysis with pyrosequencing (PSQ). After optimizing polymerase chain reaction (PCR) conditions, PCR products subjected to HRM analysis could directly be used for PSQ. By analyzing oligodeoxynucleotide controls, representing the 36 theoretically possible variant combinations for diploid human cells (8 triple-homozygous, 12 double-homozygous, 12 double-heterozygous and 4 triple-heterozygous combinations), 34 out of the 36 variant combinations could be genotyped unambiguously by combined analysis of HRM and PSQ data, compared to 22 variant combinations by HRM analysis and 16 variant combinations by PSQ. Our approach was successfully applied to genotype stable cell lines of different origin, primary human tumor cell lines from glioma patients, and breast tissue samples.

## 1. Introduction

Single nucleotide polymorphisms (SNPs) are the most abundant type of genetic variations in the human genome [1]. A detailed map of genetic variations constructed by the 1000 Genomes Project hints at more than 80 million SNPs [2]. By applying high-throughput platforms such as high-density SNP microarrays and next generation sequencing (NGS) technologies, genome-wide association studies (GWAS) have linked thousands of SNPs to multifactorial diseases [3], including rheumatoid arthritis [4], schizophrenia [5], and cancer [6,7]. More than 90% of disease-associated SNPs are located in non-coding regions, including promoter regions and enhancers. Increasing evidence suggests that sequence variants in regulatory elements may have an impact on gene expression by affecting transcription factor binding or by altering the DNA methylation status [8,9,10]. The suitability of regulatory SNPs as potential diagnostic, prognostic and/or predictive biomarker has been investigated in numerous studies [6,8,11].

Genotyping of candidate SNPs frequently involves rather cost-efficient methodologies such as allele specific polymerase chain reaction (PCR), high resolution melting (HRM) analysis, or pyrosequencing (PSQ) [12,13]. Genotyping by HRM analysis makes use of differences in the melting behavior of PCR products including the SNP of interest. After amplification of the DNA template in the presence of a saturating intercalating dye, the PCR products are subjected to an increasing temperature gradient. Melting of the double stranded amplicons results in release of the intercalating dye and thus a rapid decrease of fluorescence intensity [14]. The melting behavior depends on various parameters, including amplicon length and the content of guanine (G) and cytosine (C) [15]. HRM analysis is highly suitable for the discrimination between homozygous and heterozygous variants [16]. By a heating and cooling step prior to melting, a homozygote genotype leads to the formation of a homoduplex, whereas for a heterozygote genotype, two homoduplexes and two (mismatched) heteroduplexes are obtained. Thus, heterozygotes usually result in composite melting curves, reflecting the sum of all melting transitions. HRM analysis has frequently been used for genotyping individual SNPs [17,18,19]. However, several studies investigated its potential to genotype multiple SNPs in one and the same amplicon [18,20,21,22,23]. In principle, HRM analysis has been found to be suitable for discrimination between SNP genotype combinations. Specific variant combinations could be genotyped unambiguously, whereas others resulted in identical melting curves.

In general, PSQ is highly suitable for genotyping individual SNPs and even SNP combinations. However, PSQ doesn’t allow genotyping SNPs in homopolymeric stretches [24]. In addition, it does not provide allele specific information.

In this study, we aimed to investigate whether the power of discriminating SNP genotype combinations can be increased by coupling HRM analysis with PSQ. Since the two technologies are complementary, we hypothesized that discrimination between variant combinations for which HRM analysis does not yield unambiguous results should be possible by taking both HRM and PSQ data into account. With the aim to save time and costs and lower contamination risks, we investigated if PCR products subjected to HRM analysis can be used for PSQ. Since HRM is non-destructive, direct coupling of HRM with PSQ should, in principle, be possible. However, the two methodologies involve different chemistries. PSQ is a sequencing-by-synthesis method, in which the incorporation of the correct deoxynucleotide results in the release of pyrophosphate, and, via an enzyme cascade, in generation of a chemiluminescence signal [25]. Thus, unlike commercial PCR-HRM mixes, mixes developed for PCR and subsequent PSQ analysis do not contain an intercalating dye suitable for HRM analysis. While commercial PCR-HRM mixes are designed to obtain optimal HRM results, PCR-PSQ mixes allow achieving efficient PCR amplification and, as a consequence, high signals in PSQ. Thus, a prerequisite for coupling HRM analysis with PSQ was to optimize the composition of the PCR master mix in order to achieve satisfactory results with both methodologies. To the best of our knowledge, coupling of HRM with PSQ has been reported for bisulfite treated DNA to perform DNA methylation analysis [26] but not in the context of SNP genotyping.

For our investigations, we selected the SNPs rs527559815 (NC_000010.11: g.129467237C>T, minor allele frequency (MAF) < 0.01 (1000 Genomes Phase 3)), rs547832288 (NC_000010.11: g.129467264C>T, MAF < 0.01), and rs16906252 (NC_000010.11: g.129467281C>T, MAF 0.02), located in the promoter of the O6-methylguanine-DNA methyltransferase (*MGMT*) gene. The *MGMT* gene encodes a DNA repair protein preferentially repairing O6-methylguanine lesions. rs527559815, rs547832288, and rs16906252 are biallelic SNPs belonging to class 1 (C>T), the most frequent SNP class in the human genome.

The T allele of SNP rs16906252 has been associated with hypermethylation of the *MGMT* promoter [27,28,29]. Hypermethylation of the *MGMT* promoter is considered a predictive biomarker for the response to temozolomide (TMZ), an alkylating agent playing a crucial role in the treatment of glioblastoma multiforme (GBM) [30].

We demonstrate that under optimized conditions, PCR products subjected to HRM analysis can be used directly for PSQ. Results obtained by analyzing mixtures of oligodeoxynucleotides, representing the 36 possible variant combinations of the three SNPs, as well as DNA extracts from a variety of cell lines and tissue samples indicate that the power of discrimination between the variant combinations could be drastically increased by taking both HRM and PSQ data into account.

## 2. Results

### 2.1. Primer Design

**Figure 1:**
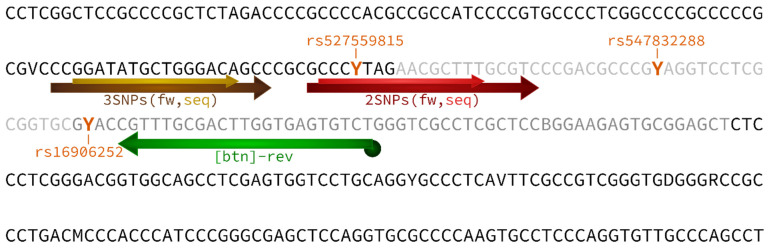
Schematic representation of part of the *MGMT* promoter region (Genbank GRCh38.p13 chr 10 NC_000010.11: range 129,467,138 to 129,467,477; 340 bp) containing the first exon, which is non-coding (marked in light gray), an enhancer element (gray, which includes the last 17 bp of exon 1), the primer binding sites (arrows), and all short variants of the 1000 Genomes project present in the Ensembl database. The 2 SNPs assay covers rs547832288 and rs16906252, and its forward (dark red) and sequencing (red) primer overlap with SNP rs527559815. The 3 SNPs assay, covering the SNPs (highlighted in orange) rs527559815 (position 129,467,237), rs547832288 (position 129,467,264), and rs16906252 (position 129,467,281), involves the forward (brown) and sequencing (ocher) primer. The biotinylated reverse primer (green) was used in both assays.

We designed a primer set, consisting of forward, reverse, and sequencing primer, aiming at genotyping the SNPs rs527559815, rs547832288, and rs16906252 in the *MGMT* promoter (Figure 1) by both HRM analysis and PSQ. We started from a primer set published previously for genotyping SNP rs16906252 by HRM analysis, resulting in a 74 bp amplicon (2 SNPs assay) [27]. At the time the primer pair was published, neither SNP rs527559815 nor SNP rs547832288 had been identified [2,27]. In order to be able to genotype each of the three SNPs, we extended the target region at the 5′ end and designed a novel forward primer, resulting in a 97 bp amplicon, and a sequencing primer for PSQ (3 SNPs assay). Since we were interested in comparing the discrimination power of both assays, we also designed a sequencing primer for the 2 SNPs assay (Figure 1). All primer sequences are given in Table 1.

### 2.2. Optimization of Coupling HRM Analysis with PSQ

First, we checked if biotinylation at the 5′-end of the reverse primer (Table 1, Figure 1), as required for PSQ, had an impact on the melting behavior of the PCR products. Biotinylation neither affected melting temperature (Tm) nor the shape of the melting curves (data not shown).

Next, we tested the suitability of two commercial PCR master mixes for HRM analysis and subsequent PSQ. The EpiTect HRM Master Mix (ET), designed for HRM analysis, was used as provided by the supplier. The PyroMark PCR Master Mix (PM), intended for PSQ, was tested in three variations: in the absence of CoralLoad Concentrate and the presence of EvaGreen dye (PMC−E+); in the presence of both CoralLoad Concentrate and EvaGreen dye (PMC+E+); and, as recommended by the supplier, in the presence of CoralLoad Concentrate and the absence of EvaGreen dye (PMC+E−).

Optimization experiments were performed on five samples, including two NHGRI samples (HG00384, NA19664), the colon cancer cell lines HCT116 and HT-29, and the non-cancerous tissue sample BRM04.

**Figure 2:**
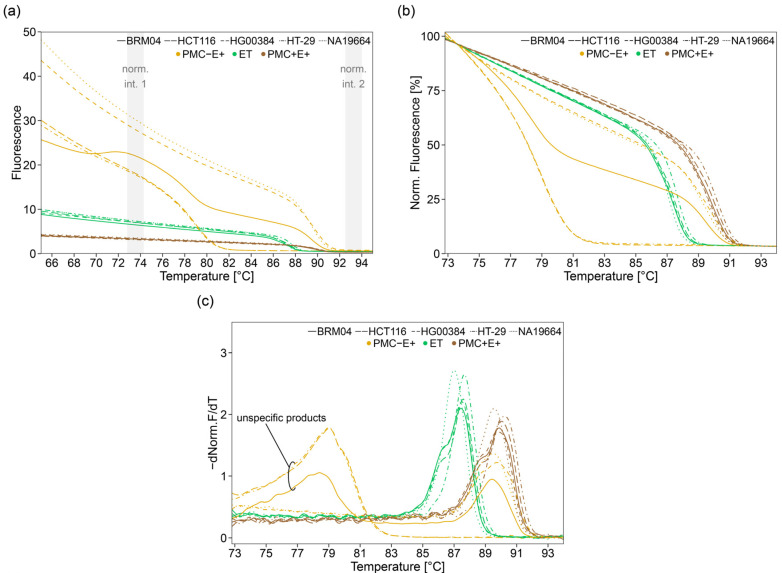

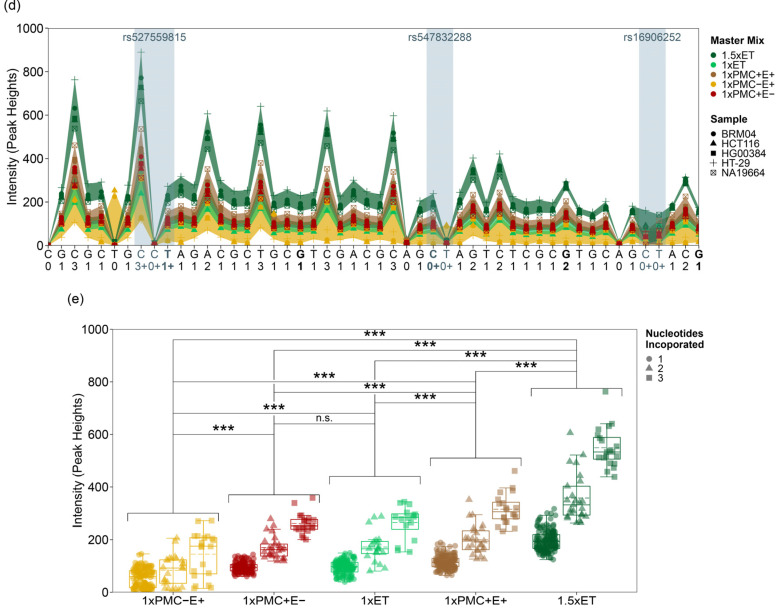
Testing the suitability of different master mixes using the 3 SNPs assay for (**a**–**c**) HRM analysis and (**d**,**e**) PSQ. Five samples, which were homozygous C for rs527559815 and rs547832288 and either homozygous C (HT-29), T (NA19664), or heterozygous (HG00384, HCT116, BRM04) for rs16906252, were selected. PyroMark PCR Master Mix (PM) with (+) or without (−) CoralLoad Concentrate (C) and EvaGreen dye (E) as well as EpiTect HRM Master Mix (ET) were used. (**a**) raw melting curves, (**b**) normalized melting curves (normalization range 1: 72.80–74.30 °C, normalization range 2: 92.50–94.00 °C) and (**c**) derivative normalized melting curves obtained by Savitzky-Golay filtering. PMC+E+ (brown), PMC−E+ (ocher) and ET (green). (**d**) Peak heights obtained from PSQ were plotted against the dispensation order and number of nucleotides incorporated; every tenth dispensation is marked in bold. Either 15 µL PCR product per 80 µL immobilization reaction (1×) or 22.5 µL PCR product per 120 µL immobilization reaction (1.5×) were used. Variable positions (additional variable nucleotide incorporation: (+)) are highlighted in blue gray. For each dispensation, the range from the lowest to the highest intensity per master mix is shaded. (**e**) Statistical comparison of peak heights between different master mixes grouped by the number of nucleotides (nts) incorporated per dispensation (1–3 fold). Variable positions were excluded; solid line represents the median, dashed line the mean and significance is marked with *** for *p* ≤ 0.001 and n.s. for not significant. All data shown originate from one and the same PCR-HRM run. For clarity reasons, the melting curve of one PCR replicate is shown.

In HRM analysis, the mix PMC−E+ resulted in about 5–8-fold higher raw fluorescence intensities within the region of normalization (72.80–74.30 °C) than the mix PMC+E+ (Figure 2a). However, in the absence of CoralLoad, amplification was unspecific, resulting in the formation of side products (~50 bp) such as primer dimers. For some samples, no specific PCR products were obtained at all (Figure 2a–c). The mix PMC+E+, containing both CoralLoad Concentrate and EvaGreen dye, resulted in melting curves characterized by low steepness at the inflection point, impairing discrimination between variant combinations, e.g., HT-29 (CCC/CCC, Tm = 90.20 °C), HG00384 (CCC/CCT, Tm = 89.70 °C and 88.80 °C), and NA19664 (CCT/CCT, Tm = 89.60 °C) (Figure 2b,c). The mix ET was found to be best suited. It resulted in about 2-fold higher raw fluorescence intensities within the normalization range than mix PMC+E+. Due to high steepness of the melting curves at the inflection point, the variant combinations CCC/CCC (HT-29, Tm = 87.70 °C), CCC/CCT (HG00384, Tm = 87.50 °C and 86.40 °C), and CCT/CCT (NA19664, Tm = 87.00 °C) could be discriminated.

Next, we tested the suitability of PCR products obtained with the master mixes PMC−E+, PMC+E−, PMC+E+, and ET for PSQ. Since PSQ is based on sequencing by synthesis, double stranded PCR products have to be separated into single strands. We applied a protocol based on streptavidin-coated sepharose beads commonly used in our lab (1×: 15 µL biotinylated PCR product, 1 µL streptavidin beads in a total volume of 80 µL). For PCR products obtained with mix ET, peak heights by consecutive incorporation of one, two or three deoxynucleotides ranged from 150 (dispensation 11 (A)) to 39 (dispensation 42 (G)), 288 (dispensation 13 (A)) to 81 (dispensation 40 (G)), and 343 (dispensation 3 (C)) to 154 (dispensation 27 (C)), respectively (Figure 2d,e). With increasing dispensation number, signals decreased about 1/3. Peak heights for dispensations 41–43, 45, 48, and 50, resulting in single nucleotide incorporation, ranged from 102 to 39, hampering genotyping of SNP rs16906252 (dispensation 46–47). Similar signal heights (*p* = 0.880) were obtained after amplification with mix PMC+E−. PCR amplification by using PMC+E+ resulted in significantly higher PSQ signals (*p* ≤ 0.001). The formation of unspecific products when using mix PMC−E+, as detected by HRM analysis, was confirmed by PSQ by the presence of a peak at dispensation 6 (0T) (Figure 2d and Figure S1).

Since mix ET was found to be best suited for HRM analysis, we attempted to enhance its applicability for PSQ. In particular, we tried to achieve higher signals at higher dispensation numbers in order to increase accuracy of genotyping SNP rs16906252 by further optimizing immobilization of biotinylated PCR products to streptavidin-coated sepharose beads. First, we varied the ratio of PCR product to streptavidin-coated sepharose beads. Neither decreasing (5 µL, 7.5 µL) nor increasing (22.5 µL, 30 µL, and 39 µL) the amount of biotinylated PCR product resulted in both, higher signals at high dispensation numbers and more accurate genotyping of SNP rs16906252 (Figure S2). However, by upscaling the immobilization reaction by a factor of 1.5 (22.5 µL biotinylated PCR product, 1.5 µL streptavidin beads in a total volume of 120 µL), significantly higher signals (*p* ≤ 0.001) were achieved compared to our commonly used protocol (Figure 2e). Consecutive incorporation of one, two, or three deoxynucleotides led to signals ranging from 317 to 125, 606 to 263, and 763 to 438, respectively (Figure 2d,e).

Due to signal decrease of about 1/3, peak heights for dispensations 41–43, 45, 48, and 50 were between 202 and 125. As a consequence, SNP rs16906252 could be genotyped with higher accuracy.

Since these results also hold true for the 2 SNPs assay, all following experiments were performed using mix ET for PCR amplification. For subsequent PSQ, the 1.5× upscaled immobilization protocol was applied.

### 2.3. Discrimination Power of the 2 SNPs Assay

The discrimination power of the 2 SNPs assay was determined by analyzing mixtures of oligodeoxynucleotides, representing the 36 possible variant combinations of the three C>T SNPs rs527559815, rs547832288, and rs16906252 (8 triple-homozygous, 12 double-homozygous, 12 double-heterozygous and 4 triple-heterozygous combinations; Supplementary Table S1). In order to investigate the applicability for real samples, we also analyzed NHGRI samples for which the genotypes at the three SNP loci were available.

Although the SNP rs527559815 was located in the binding site of the forward primer, containing a C at position rs527559815, PCR products were obtained for all variant combinations. However, we observed an increase of Ct values from variant combinations being homozygous C at rs527559815 to heterozygous variant combinations for rs527559815 to variant combination being homozygous T at rs527559815. For example, the amplification curves of double-heterozygous combinations ((CCC/TTC (Ct value: 30.61), TCC/CTC (Ct value: 30.98)) were in between those of the triple-homozygous combinations consisting of the respective DNA strand of the C allele of rs527559815 (CCC/CCC (Ct value: 29.91), CTC/CTC (Ct value: 29.81)) and those consisting of the respective DNA strand carrying the T allele (TTC/TTC (Ct value: 32.36) or TCC/TCC (Ct value: 32.78)), indicating a PCR bias of the DNA strand having the C allele of rs527559815.

**Figure 3:**
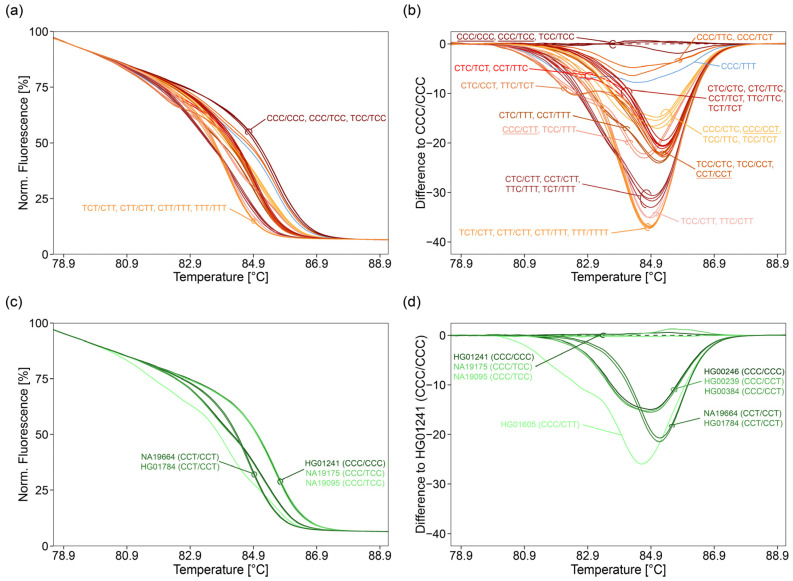
Genotype discrimination of the 2 SNPs assay by HRM analysis. Data are shown as (**a**,**c**) normalized melting curves and (**b**,**d**) difference to the triple-homozygous control CCC/CCC of the respective sample set (dashed line). (**a**,**b**) Only one oligodeoxynucleotide control (blue, CCC/TTT) had a distinguishable melting curve. Manual evaluation of the data obtained for the remaining 35 oligodeoxynucleotide controls resulted in 12 groups of melting curves (orange/red). (**c**,**d**) Application to NHGRI samples. The melting curve of HG00246, indicated as CCC/CCC in the data base, grouped together with those of HG00384 (CCC/CCT) and HG00239 (CCC/CCT). Data displayed in one plot originated from the same PCR-HRM run. For clarity reasons, the melting curve of one PCR replicate is shown.

By HRM analysis, only one of the 36 oligodeoxynucleotide controls, the triple-heterozygous combination CCC/TTT, was unambiguously identified (Figure 3a,b). The remaining 35 variant combinations resulted in 12 groups of melting curves (six groups containing two, two groups containing three, three groups containing four, and one group containing five variant combinations).

Due to the PCR bias, melting curves of double-heterozygous combinations being heterozygous for rs527559815 (e.g., CCC/TTC and TCC/CTC) were different from those obtained for the respective triple-homozygous combinations (CCC/CCC and TTC/TTC, or CTC/CTC and TCC/TCC).

Despite this bias, three of four pairs of the double-heterozygous combinations being heterozygous for rs527559815, having one identical DNA strand, and being reciprocal (C and T, or T and C) at loci rs547832288 and rs16906252 could not be distinguished: CCC/TTC and CCC/TCT, TCC/CTC and TCC/CCT, CTC/TTT and CCT/TTT) (Figure 3b). However, the double-heterozygous combination TCT/CTT was in another group of melting curves than the double-heterozygous combination TTC/CTT and the triple-heterozygous combination TCC/CTT.

Variant combinations being homozygous at rs527559815, homozygous T at either rs547832288 or rs16906252, and heterozygous at the other locus, could not be distinguished, e.g., CTC/CTT, CCT/CTT, TTC/TTT, and TCT/TTT. This also hold true for variant combinations being either heterozygous or homozygous C for rs547832288 and rs16906252. In addition, variant combinations leading to complex melting curves could not be distinguished, in case they were reciprocal heterozygous at rs547832288 and rs16906252, and heterozygous at rs527559815, e.g., CTC/TCT and CCT/TTC, or homozygous at rs527559815, e.g., CTC/CCT and TTC/TCT, or CCC/CTT and TCC/TTT. Variant combinations, which were homozygous C for both rs547832288 and rs16906252, could not be distinguished. The same was true for variant combinations being homozygous T at both rs547832288 and rs16906252, e.g., CTT/CTT, CTT/TTT, and TTT/TTT. In addition, melting curves of some variant combinations grouped together, e.g., CCT/CCT with TCC/CTC and TCC/CCT. The triple-homozygous combinations CTC/CTC, TTC/TTC, and TCT/TCT and the double-homozygous combinations CTC/TTC and CCT/TCT had identical melting curves with a single melt transition.

**Figure 4:**
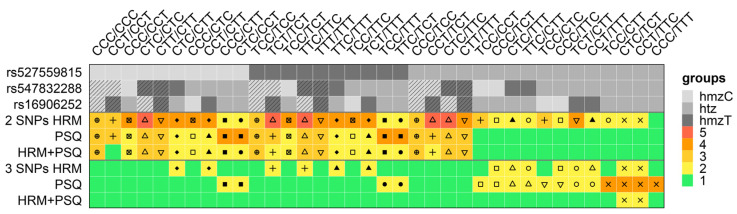
Comparison of genotype discrimination between the 2 SNPs and 3 SNPs assay, by taking results from HRM analysis and PSQ into account either separately or together (HRM+PSQ). Genotypes of the SNPs from the 36 oligonucleotide controls are shaded in gray for homozygous C (light), homozygous T (dark) and heterozygous C/T or T/C (medium). Variants being homozygous at both loci, rs547832288 and rs16906252, are additionally shaded with hatched areas. Numbers (1–5)/colors (green to orange/red) refer to the discrimination power. 1 (green) indicates that the variant combination was unambiguously identified. 2–5 (yellow to orange/red) indicates that the group the variant combination was assigned to contain from 2 to 5 members, respectively. Members of one group are marked by the same symbol.

In addition to Ct values and melting curves, the pyrograms indicated that amplification at the rs527559815 locus was biased to the C allele. C/T ratios for heterozygous variable positions of double- and triple-heterozygous variant combinations that were heterozygous for rs527559815 were altered compared to the homozygous variant combinations. For example, C/T ratio for CTC/TCT was decreased at rs547832288 and increased at rs16906252 compared to CTC/CCT, TTC/TCT, and CCT/CCT (Figure S3). C/T ratio for CCT/ TTC was increased at rs547832288 and decreased at rs16906252 compared to CTC/CCT, TTC/TCT, and CCT/CCT. Due to these altered C/T ratios, the twelve variant combinations could be distinguished by manual evaluation of PSQ data (Figure 4, lane: 2 SNPs PSQ). All variant combinations that are homozygous for rs527559815 could not be unambiguously determined. In addition, variant combinations which were heterozygous at rs527559815 and homozygous at both, rs547832288 and rs16906252, could not be distinguished from the respective triple-homozygous variant combinations, e.g., CCT/CCT, TCT/TCT, and CCT/TCT (Figure 4, lane: 2 SNPs PSQ).

Thirteen out of 36 variant combinations could be distinguished by taking data from both HRM analysis and PSQ into account. However, the remaining 23 variant combinations could not be genotyped unambiguously (Figure 4, lane: 2 SNPs HRM+PSQ).

When we applied the 2 SNPs assay to nine NHGRI samples, representing five variant combinations (CCC/CCC, CCC/TCC, CCC/CCT, CCT/CCT, and CCC/CTT), four groups of melting curves were obtained (Figure 3d), because discrimination between CCC/CCC and CCC/TCC is not possible. In concordance with results obtained for oligodeoxynucleotide controls (Figure 3b), none of the samples could be genotyped unambiguously. However, with the exception of sample HG00246, the group the sample was assigned to, included the variant combination given in the Ensembl data base. For sample HG00246 (CCC/CCC), the variant combination was found to be either CCC/CTC, CCC/CCT, TCC/TTC, or TCC/TCT.

PSQ did also not allow unambiguous genotyping of the nine NHGRI samples (Figure 4, lane: 2 SNPs PSQ). The variant combination given in the Ensembl data base was one of the possible combinations found by PSQ, except for sample HG00246. PSQ results for sample HG00246 suggest the genotype to be either CCC/CCT or TCC/TCT (data not shown).

Only by combined evaluation of HRM and PSQ data, the variant combination CCT/CCT (samples HG01784 and NA19664) could be genotyped correctly (Figure 4, lane: 2 SNPs HRM+PSQ). For sample HG00246 (CCC/CCC), combined analysis gave a genotype of either CCC/CCT or TCC/TCT.

### 2.4. Discrimination Power of the 3 SNPs Assay

**Figure 5:**
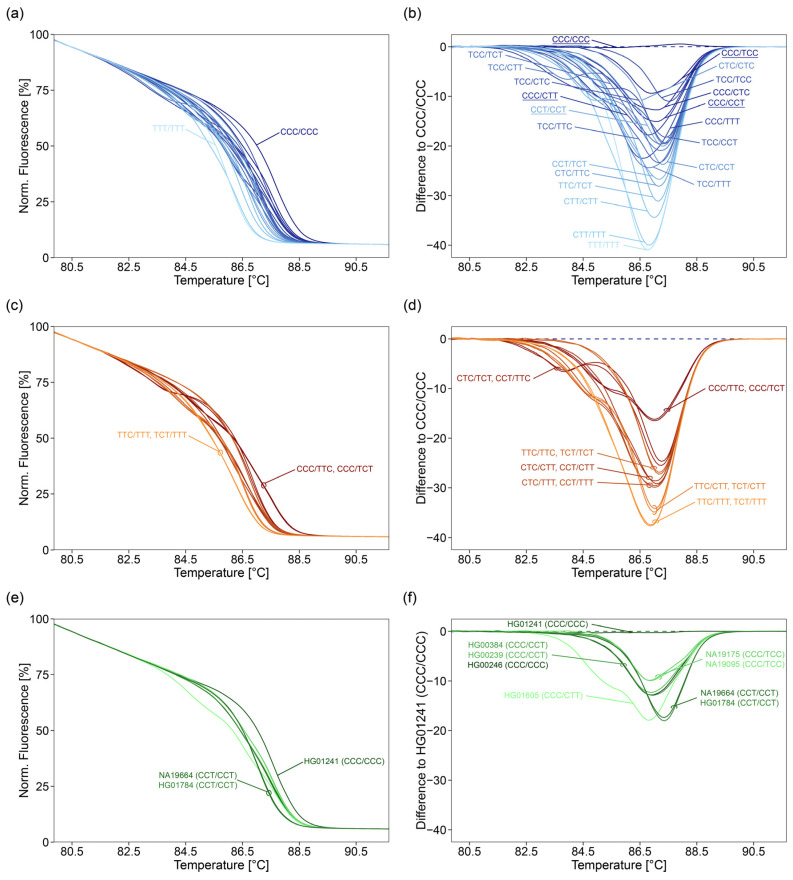
Genotype discrimination of the 3 SNPs assay by HRM analysis. Data are shown as (**a**,**c**,**e**) normalized melting curves and (**b**,**d**,**f**) difference to the triple-homozygous control CCC/CCC of the respective sample set (dashed line). (**a**,**b**) 22 diploid oligonucleotide controls that could be distinguished manually included all 5 variant combinations (underlined) present in the NHGRI Sample Repository for Human Genetic Research. (**c**,**d**) 14 diploid oligonucleotide controls grouped in 7 pairs of identical melting curves. (**e**,**f**) The melting curve of NHGRI sample HG00246, indicated as CCC/CCC in the Ensembl data base, grouped together with those of HG00384 (CCC/CCT) and HG00239 (CCC/CCT). Data displayed in one plot originate from the same PCR-HRM run. For clarity reasons, the melting curve of one PCR replicate is shown.

Analogue to the 2 SNPs assay, discrimination power of the 3°SNPs assay was investigated by analyzing the 36 oligodeoxynucleotide controls and the NHGRI samples (Figure 4, lanes: 3 SNPs, Figure 5a–f).

22 oligodeoxynucleotide controls (6 triple-homozygous, 8 double-homozygous, 6 double-heterozygous, and 2 triple-heterozygous) were accurately identified by unique melting curves (Figure 5a,b). The remaining 14 controls (CTC/TCT, CCT/TTC; CCC/TTC, CCC/TCT; TTC/TTC, TCT/TCT; CTC/CTT, CCT/CTT; CTC/TTT, CCT/TTT; TTC/CTT, TCT/CTT; TTC/TTT, TCT/TTT) led to pairwise identical melting curves (Figure 5c,d).

All triple-homozygous combinations had a single melting transition except CCT/CCT (Figure S4a). The derivative melting curve of CCT/CCT showed an additional small shoulder at 85.76 °C, allowing discrimination between CCT/CCT and CTC/CTC. Corresponding triple-homozygous combinations being homozygous T at locus rs527559815 (TTC/TTC and TCT/TCT) could not be discriminated from each other as both had a single melt transition at 86.76 °C. A single T in triple-homozygous combinations decreased Tm by 0.4 °C (rs527559815), 0.7 °C (rs547832288), or 0.6 °C (rs16906252), compared to the variant combination CCC/CCC. The occurrence of two Ts, one at rs527559815 and the other one at either rs547832288 or rs16906252, resulted in a decrease of 1.0 °C. The triple-homozygous combination having two Ts, one at rs547832288 and one at rs16906252, and the triple-homozygous combination having three Ts led to a decrease of 1.3 °C and 1.7 °C, respectively.

Double-homozygous combinations showed two melting transitions, leading to derivative melting curves containing shoulders (Figure S4b). The difference between peak and shoulder was about 1.0 °C, except for the variant combination CCC/CTC (1.4 °C). The variant combinations being homozygous either C or T for rs527559815, homozygous T at rs547832288 or rs1690625, and reciprocal heterozygous at the other locus (rs547832288 or rs1690625), had derivative curves with identical Tms and peak height ratios, e.g., CTC/CTT and CCT/CTT. In contrast to the other variant combinations, TTC/TTT and TCT/TTT only had a slight shoulder at the derivative curve.

Half of the double-heterozygous combinations had two individual melting transitions, with a difference in Tm of about 2.2–2.4 °C. The variant combinations TTC/CTT and TCT/CTT resulted in derivative curves with identical Tms and peak height ratios (Figure S4c). Double-heterozygous combinations having either CCC or TTT in one DNA strand yielded three melting transitions. Three peak maxima were obtained for the distinguishable variant combinations CCC/CTT and TCC/TTT. Identical melting curves containing two peak maxima and one shoulder were obtained for the pairs CCC/TTC and CCC/TCT, and CTC/TTT and CCT/TTT.

Among the triple-heterozygous combinations, the melting curve of CCC/TTT was most complex, having three main melting transitions (Tm: 87.56 °C, 85.96 °C, and 83.06 °C), and two minor transitions causing peak shoulders (Tm: 83.96 °C and 81.86 °C) (Figure S4d). The combination TCC/CTT had one melting transition at 83.16 °C and two transitions at 87.16 °C and 86.66 °C, which led to a broad double-shouldered peak. The variant combinations CTC/TCT and CCT/TTC had identical melting curves with three melting transitions, which led to one peak at approximately 86.86 °C, and a second peak with a shoulder at approximately 83.36 °C and 82.56 °C, respectively.

Since PSQ is not allele specific, 16 out of 36 variant combinations could not be discriminated from each other (Figure 4, lane: 3 SNPs PSQ), including four triple-heterozygous variant combinations (TCC/CTT, CTC/TCT, CCT/TTC, and CCC/TTT) and six pairs of double-heterozygous variant combinations (CCC/CTT, CTC/CCT; TCC/TTT, TTC/TCT; TCC/CCT, CCC/TCT; CTC/TTT, TTC/CTT; TCC/CTC, CCC/TTC; and TCT/CTT, CCT/TTT).

However, by combined analysis of HRM and PSQ data, 34 out of 36 variant combinations could be genotyped unambiguously (Figure 4, lanes: 3 SNPs). The triple-heterozygous combinations CTC/TCT and CCT/TTC were the only ones that could not be distinguished from each other.

The melting curves (Figure 5a,b,e,f) obtained for the nine NHGRI samples were similar to those obtained for respective oligodeoxynucleotide controls (CCC/CCC, CCC/TCC, CCC/CCT, CCT/CCT, and CCC/CTT). NHGRI samples differing in variant combination could be distinguished by HRM analysis. With the exception of sample HG00246, the variant combination given in the Ensembl data base was determined (Figure 5f). For sample HG00246 (CCC/CCC), the variant combination CCC/CCT was obtained.

By PSQ, all NHGRI samples except HG01605 could be genotyped unambiguously (Figure 4, lane: 3 SNPs PSQ). For sample HG01605 (CCC/CTT), the variant combination was found to be either CCC/CTT or CTC/CCT. The variant combination given in the Ensembl data base was found for all samples, except for sample HG00246. In line with HRM results, the variant combination CCC/CCT was found for sample HG00246 (CCC/CCC) (Figure S5).

Unambiguous genotyping of HG01605 (CCC/CTT) was possible by combined evaluation of HRM and PSQ data (Figure 4, lane: 3 SNPs HRM+PSQ).

### 2.5. Application of the 3°SNPs Assay to Cell Lines

**Figure 6:**
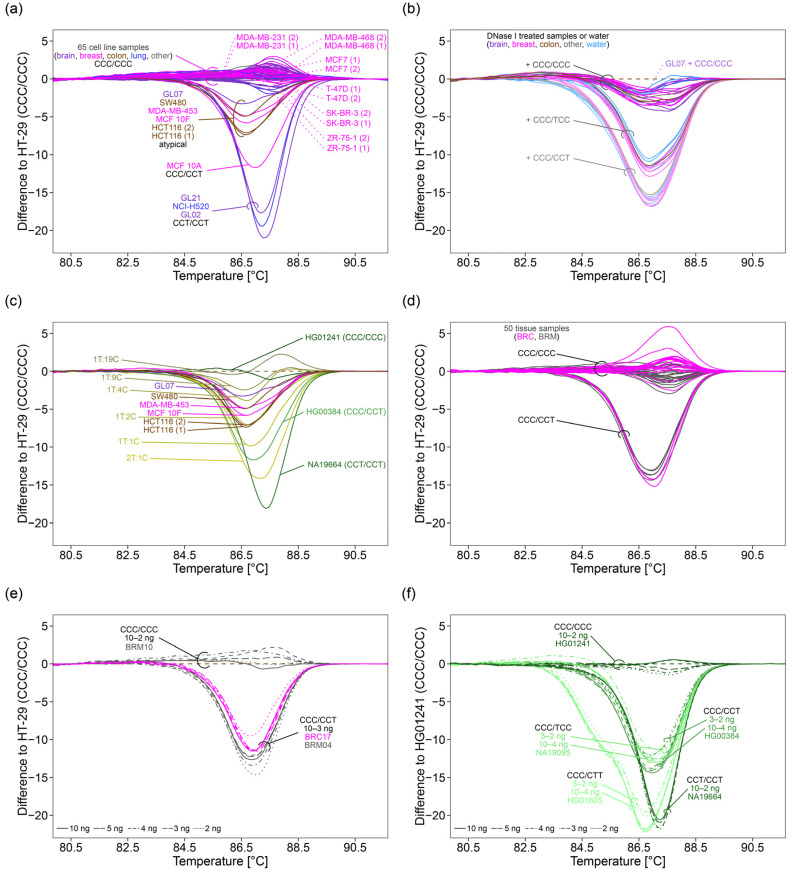
Application of the 3 SNPs assay by HRM analysis on biological samples shown as difference to the triple-homozygous control CCC/CCC of the respective sample set (straight dashed line). (**a**) In 75 cell line samples originating from brain (purple), breast (pink), colon (brown), lung (blue) and other tissues (black), the genotypes CCC/CCC (dark), CCC/CCT (lighter) and CCT/CCT (light) and a group with atypical melting curves between CCC/CCC and CCC/CCT were found. (**b**) Oligonucleotide controls (CCC/CCC (dark), CCC/TCC (lighter), CCC/CCT (light) added to DNase I treated samples (n = 17, n = 6, n = 6, respectively, colored by tissue origin) grouped together with the respective oligonucleotide control added to water (gray, n = 5). (**c**) NHGRI sample mixtures of NA19664 (CCT/CCT) and HG01241 (CCC/CCC) in ratios from 1T:2C to 1:4 (1T:4C) and cell lines having atypical melting curves had similar melting curve shapes. (**d**) In breast cancer (pink) and normal control tissue (gray), the genotypes CCC/CCC (dark) and CCC/CCT (lighter) were found and (**e**) could be distinguished down to 3 ng. (**f**) All 5 variant combinations present in the NHGRI Sample Repository for Human Genetic Research could be distinguished down to 4 ng. (**e**,**f**) 10 ng (solid), 5 ng (long-dashed), 4 ng (dashed), 3 ng (dot-dashed), and 2 ng (dotted) initial DNA amount for PCR are shown. (**a**,**b**,**d**,**e**) Curves shown in one plot originate from multiple runs. For clarity reasons, the melting curve of one PCR replicate is shown.

Aiming at investigating the applicability and robustness of the 3°SNPs assay, 33 human stable cell lines originating from brain, breast, cervix, colon, lung, peripheral blood, and skin cancer and breast fibrocystic disease were analyzed. In addition, the 3°SNPs assay was applied to 35 primary human tumor cell lines established from 26 glioma patients. PCR amplification was successful for all cell line samples. By HRM analysis, the variant combination CCC/CCC was determined for 27 stable cancer cell lines and 32 primary glioma cell lines (Figure 6a, Supplementary Table S2). The stable cell line MCF 10A was genotyped CCC/CCT. Melting curves characteristic for the variant combination CCT/CCT were obtained for one stable cell line (NCI-H520) and two primary glioma cell lines (GL02 and GL21). By PSQ, the variant combinations determined by HRM analysis could be confirmed.

However, four stable cancer cell lines (SW480, MDA-MB-453, MCF 10F, and HCT116) and one primary glioma cell line (GL07) resulted in a group of atypical melting curves, being in between the curves obtained for CCC/CCC and CCC/CCT. In addition, irregular pyrograms were obtained for these samples (Figure S6). While rs527559815 and rs547832288 were unambiguously genotyped C/C, respectively, warning messages like “uncertain” or “failed genotype determination” were obtained for rs16906252. rs16906252 was genotyped either C/C or C/T. Even for two technical replicates, different results were obtained (Figure S6).

### 2.6. Elucidating the Cause of Atypical Melting Curves and Irregular Pyrograms

In order to investigate if the atypical melting curves and irregular pyrograms were caused by matrix effects, e.g., by residues from specific cell culture conditions and/or specific DNA extraction kits, we compared the results obtained for DNA extracts from seven cell lines (MCF7, MDA-MB-231, MDA-MB-468, SK-BR-3, T-47D, ZR-75-1, and HCT116). These cell lines were selected because they were handled in different labs and thus were cultured under different conditions and/or were subjected to different DNA extraction protocols. A DNA extract obtained by using the AllPrep DNA/RNA Mini Kit (Qiagen, Hilden, Germany) was available for each of the cell lines. ZR-75-1 and HCT116 had been cultured under similar conditions, whereas MDA-MB-231, MDA-MB-468, SK-BR-3, and T-47D were cultured in one lab with RPMI-1640 medium, and in the other lab with either DMEM/high, L-15 Leibovitz or MEM medium (Supplementary Table S2). We did not find the occurrence of atypical melting curves to be associated with cell culturing conditions or DNA extraction kit (Figure 6a, melting curves from different DNA extracts were marked with (1) and (2)).

With PSQ, DNA extracts from one and the same cell line obtained by specific cell culture conditions and/or specific DNA extraction protocols were investigated for MDA-MB-231, MDA-MB-468, and HCT116. DNA extracts from MDA-MB-231 and MDA-MB-468 yielded regular pyrograms, whereas for HCT116 irregular pyrograms were obtained (Supplementary Table S2), indicating that the occurrence of irregular pyrograms was not associated with cell culturing conditions or the DNA extraction kit.

To further elucidate the impact of matrix effects on the melting behavior of PCR products, we designed a DNase I treatment protocol for digesting DNA in DNA extracts and applied it to 15 cell lines determined to have the variant combination CCC/CCC (HeLa, HT-29, BT-20, HCC1937, HMEC, Hs 578T, MCF7 (grown in DMEM media), MDA-MB-231 (both DNA extracts), A431, GL01, GL06, GL08c, GL11, GL19e, and GL23b), and to GL07 yielding an atypical melting curve and an irregular pyrogram (Supplementary Table S2).

After DNA digestion, the oligodeoxynucleotide control CCC/CCC was added to each of the 17 DNase I treated DNA extracts. The oligodeoxynucleotide controls CCC/TCC or CCC/CCT were added to a subset (HT-29, BT-20, HCC1937, Hs 578T, MCF7, and MDA-MB-231 (2)). The shape of the melting curves obtained after DNA digestion and spiking only slightly differed from those obtained by repeated analysis of the respective oligodeoxynucleotide control in water (Figure 6b). For none of the spiked DNase I treated DNA extracts, atypical melting curves were observed, suggesting that the atypical shape of the melting curves was not caused by matrix effects.

Thus, we investigated if aberrant copy numbers could be responsible for the atypical melting curves obtained by the 3 SNPs assay. Since irregular pyrograms indicated the presence of one DNA strand with CCT and/or one DNA strand with CCC, we diluted CCT in CCC by mixing the NHGRI samples NA19664 (CCT/CCT) and HG01241 (CCC/CCC) in ratios from 2:1 (2T:1C) to 1:19 (1T:19C). With increasing amounts of CCC, the formation of less heteroduplexes and more homoduplexes altered the shape of melting curves. The peak shoulder height of the derivative melting curve decreased from 2.15 (2T:1C) to 1.24 (1T:10C) and the peak height increased from 2.49 (2T:1C) to 3.00 (1T:19C) (not shown).

The shape of melting curves obtained for sample HG00384 (CCC/CCT) (shoulder to peak ratio: 0.76) and for 1T:1C (shoulder to peak ratio: 0.71) was similar (Figure 6c). The atypical melting curves obtained for HCT116 (shoulder to peak ratios: 0.61 and 0.59) corresponded to that of 1T:2C (shoulder to peak ratio: 0.58). Melting curves obtained for cell lines MCF 10F (shoulder to peak ratio: 0.52), MDA-MB-453 (shoulder to peak ratio: 0.49), and SW480 (shoulder to peak ratio: 0.54) were in between those obtained for 1T:2C (shoulder to peak ratio: 0.58) and 1T:4C (shoulder to peak ratio: 0.49). The melting curve obtained for GL07 (shoulder to peak ratio: 0.42) was in between those of 1T:4C (shoulder to peak ratio: 0.49) and 1T:9C (shoulder to peak ratio: 0.42) (Figure 6c).

By PSQ, a regular pyrogram was obtained for the 1T:1C mixture. PSQ of mixture 2T:1C and mixtures 1T:2C to 1T:19C led to irregular pyrograms, having altered C/T signal height ratios at variable position rs16906252. C/T signal height ratios increased with increased dilution of the CCT strand (Figure S7a). For mixtures 1T:2C to 1T:9C, “uncertain” or “failed genotype determination” warning notes were obtained, similar to pyrograms for cell lines HCT116, MCF 10F, MDA-MB-453, SW480, and GL07 (Figure S7). C/T signal height ratios were similar for the cell line HCT116 and the 1T:2C mixture. The C/T signal height ratios for the cell lines MCF 10F, MDA-MB-453, SW480, and GL07 were between those obtained for the 1T:2C and 1T:4C mixtures. Notably, from mixture 1T:2C to mixture 1T:4C, the PSQ result obtained for rs16906252 changed from C/T to C/C (Figure S7). This might be the explanation why technical replicates for cell lines resulting in irregular pyrograms (Figure S6b) yielded different results (CCC/CCT or CCC/CCC).

### 2.7. Application of the 3°SNPs Assay to Breast Tissue Samples

Next, we applied the 3°SNPs assay to 50 breast tissue samples from cancer (BRC) and non-cancerous (BRM) patients.

HRM analysis identified the variant combination CCC/CCC for 21 BRC and 21 BRM patients. The variant combination CCC/CCT was found for three BRC and five BRM patients (Figure 6d). A subset of 19 samples genotyped CCC/CCC and all eight samples genotyped CCC/CCT were subjected to PSQ. Genotypes determined by PSQ were in line with those identified by HRM analysis (Supplementary Table S3). None of the samples resulted in atypical melting curves or irregular pyrograms. However, tumor samples (pink) caused larger variation between melting curves than samples from healthy controls (gray) (Figure 6d).

### 2.8. Determination of the Minimal Initial DNA Amount

Tissue samples obtained from biopsies are often limited in amount and thus, we were interested in the minimal initial DNA amount required for accurate genotyping. By HRM analysis, CCC/CCT could be identified in breast tissue samples down to 3 ng DNA (quantified fluorometrically with the Qubit instrument) (Figure 6e). Identification of the triple-homozygous variant combination CCC/CCC was even not affected by further dilution (data not shown). However, for NHGRI samples, accurate determination of the double-heterozygous variant combination CCC/CTT and discrimination between the double-homozygous variant combinations CCC/CCT and CCC/TCC was only possible down to an initial DNA amount of 4 ng (Figure 6f). Identification of homozygous variants was not affected by a lower initial DNA amount. By PSQ, genotypes could accurately be identified down to an initial DNA amount of 1 ng (Figure S8). Input of 0.1 ng DNA caused amplification of only one DNA strand, while 0.01 ng input was too low to obtain any PCR products.

### 2.9. Cluster Analysis for Genotyping

*k*-means clustering was performed with HRM and PSQ data obtained by the 3 SNPs assay. In order to subject HRM data from multiple runs to cluster analysis, difference curves to the respective run control were used. Due to small differences in raw fluorescence intensities between different runs, the use of normalized melting curves resulted in less accurate clustering.

When analyzing both technical replicates of the 14 oligodeoxynucleotide controls with pairwise identical melting curves, direct *k*-means clustering resulted in seven clusters. These clusters corresponded with the seven groups obtained by visual evaluation of the melting curves (Figure 5d). We also investigated if data reduction by PCA prior to *k*-means clustering is of advantage. By applying PCA prior to *k*-means clustering, eleven PCs had an eigenvalue higher 1 and were explaining 96.3% of the variance. This approach resulted in either six or seven clusters. In case of obtaining six clusters, five clusters corresponded with the five groups identified by visual evaluation, and one cluster contained the group CTC/TTT and CCT/TTT and the group TTC/CTT and TCT/CTT. In case of the formation of seven clusters, four were identical with the groups obtained by visual evaluation. The fifth cluster comprised the group CTC/TTT and CCT/TTT and the group TTC/CTT and TCT/CTT. However, cluster six and seven, each containing one technical replicate of TTC/TTT and TCT/TTT, were incorrect.

For the nine NHGRI samples, five clusters were obtained by direct clustering, in accordance to the groups found by visual evaluation (Figure 5f, Supplementary Table S4). Using nine PCs (reaching an eigenvalue of 1.52 and a cumulative variance of 97.3%) also resulted in five clusters. Three of them were identical with the groups obtained by visual evaluation, while the fourth was incorrect, consisting of both technical replicates of sample NA19095 (CCC/TCC) and one technical replicate of sample NA19175 (CCC/TCC). The other replicate of sample NA19175 clustered together with HG00384 (CCC/CCT), HG00239 (CCC/CCT), and HG00246 (CCC/CCC) in the fifth cluster, which was also not correct.

In summary, clustering after PCA was less accurate than direct clustering when compared to visual evaluation. Thus, direct *k*-means clustering of HRM data was performed on difference melting curves.

For cell line samples, three clusters (CCC/CCC, atypical melting curves and CCC/CCT, and CCT/CCT) were obtained (Supplementary Table S2). Samples yielding atypical melting curves and MCF 10A, the only representative of CCC/CCT, clustered together. Analysis of oligodeoxynucleotide controls added to DNase I treated cell lines resulted in three clusters (CCC/CCC, CCC/TCC, and CCC/CCT). Clustering of mixtures of NHGRI samples and cell lines yielding atypical melting curves led to cluster 1 (NA19664 (CCT/CCT)), cluster 2 (HG00384 (CCC/CCT), 2T:1C, and one technical replicate of 1T:1C), cluster 3 (one technical replicate of 1T:1C 1T:2C, HCT116 (1), HCT116 (2), and MCF 10F), cluster 4 (1T:4C, MDA-MB-453, SW480, GL07), and cluster 5 (1T:9C, 1T:19C, and HG01241 (CCC/CCC)).

For tissue samples, two clusters were obtained, one containing samples of the genotype CCC/CCT (BRC09, BRC13, BRC17, BRM04, BRM08, BRM09, BRM13, and BRM19) and one containing the remaining tissue samples with genotype CCC/CCC (Supplementary Table S3).

For statistical evaluation and/or multidimensional representation of PSQ data for all three SNPs at once, data were normalized. The difference between the normalized peak heights of C and T was calculated and then scaled from 0 to 1 for each variable position, respectively. *k*-means clustering applied to oligodeoxynucleotide controls and biological samples analyzed by PSQ resulted in nine clusters. Six individual clusters were obtained for the variant combinations CCC/CCC, CCC/CTT, CCT/CCT, CCC/TCC, and CCC/CCT and for the samples with atypical melting curves. However, because of the low number of representatives, some of the oligodeoxynucleotide controls were found in these individual clusters (e.g., TCC/TCC in the CCC/TCC cluster) or in one of three additional clusters containing only oligodeoxynucleotide controls.

**Figure 7:**
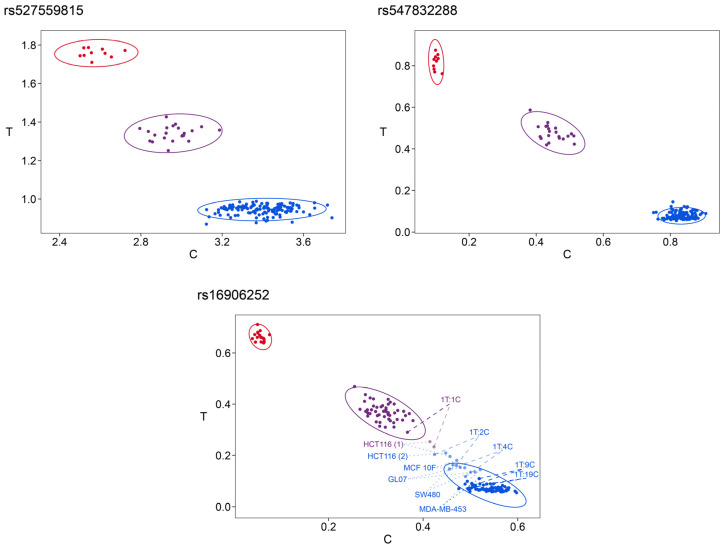
Cluster analysis of genotypes for each individual SNP obtained by PSQ. Peak heights of variable positions were normalized and given as ratios to the first 12 dispensations where one nucleotide was incorporated. Genotypes clustered for T/T (red), C/T (purple), and C/C (blue) for all three SNPs, respectively (95% confidence interval). When using four clusters for analyzing rs16906252, cell line samples and NHGRI sample mixtures of NA19664 (CCT/CCT) and HG01241 (CCC/CCC), which also had atypical melting curves, clustered together in a fourth cluster (shown in lighter colors).

Information on the respective genotypes was maintained if clustering was performed for each variable position individually (Figure 7). In contrast to cluster analysis for multiple variable positions simultaneously, cluster analysis of individual variable positions did not require scaling of normalized peak height ratios between 0 and 1. For each SNP, three clusters were obtained, each representing one of the genotypes C/C, C/T, and T/T. A fourth cluster was found for rs16906252 containing the samples with atypical melting curves and the mixtures between 1T:4C and 1T:1C marked in Figure 7.

## 3. Discussion

For investigation of the potential of HRM analysis coupled with PSQ to genotype multiple SNPs in one and the same amplicon, we selected the SNPs rs527559815, rs547832288, and rs16906252 due to the following reasons. The three SNPs are in close proximity within a region of 45 bp. Each of the SNPs is a C>T SNP, allowing us to investigate the potential of discriminating reciprocal variant combinations according to their position. The three SNPs are located in a differentially methylated region (DMR) of the *MGMT* promoter, with rs527559815 being upstream of (non-coding) exon 1, rs547832288 located in exon 1, and rs16906252 occurring in an intra-promoter enhancer element [31]. The interaction of both genetic and epigenetic alterations in regulatory regions of tumor suppressor genes and oncogenes is known to play a major role in the development and progression of cancer. There is increasing evidence, that the minor T allele of SNP rs16906252 is associated with *MGMT* promoter methylation and/or longer overall survival in colon cancer [28,32], glioma [29,33], lung cancer [27,34], and oral lichen planus [35].

An HRM method for genotyping rs16906252 has already been published [27]. However, the forward primer was not suitable for our study because its binding site overlapped with SNP rs527559815, identified after the publication date of the study by Kristensen et al. In order to be able to genotype the three SNPs of interest, we had to extend the target region and to design a novel forward primer. Many papers have shown that the sensitivity of HRM analysis decreases with increasing amplicon length [14,15,22,23]. Thus, we aimed at designing primers resulting in an amplicon as short as possible. Since we were interested in comparing the discrimination power of the assay published previously (“2 SNPs assay”) and the assay developed in this study (“3 SNPs assay”), we designed two sequencing primers, one for each assay. However, placing of primers was limited due to additional SNPs occurring in the *MGMT* promoter region.

One of our goals was to use PCR products subjected to HRM analysis directly for PSQ. In principle, direct coupling of HRM and PSQ should be possible, because HRM is a non-destructive methodology. However, some optimization was required because PCR mixes for HRM analysis and mixes for PSQ differ in their composition. PCR-HRM mixes contain an intercalating fluorescent dye and are optimized to obtain high fluorescence-signal-to-noise-ratio, high difference between melting temperature of homozygous variants, and heteroduplex formation for heterozygous variants. In contrast, commercial PCR mixes for subsequent PSQ analysis do not contain an intercalating fluorescent dye suitable for HRM analysis and are optimized to achieve high PCR product yield in order to obtain high signal-to-noise-ratios even at high dispensation number. We tested two commercial mixes for their potential to yield good results in both HRM analysis and PSQ, the EpiTect HRM Master Mix (ET) for the Rotor-Gene Q device, containing EvaGreen dye, and the PyroMark PCR Master Mix (PM), generally applied for PSQ with PyroMark instruments, not containing EvaGreen dye (E−). The PM mix was tested in three versions, in the absence of CoralLoad Concentrate and the presence of EvaGreen dye (PMC−E+); in the presence of CoralLoad Concentrate and the absence of EvaGreen dye (PMC+E−); and in the presence of both CoralLoad Concentrate and EvaGreen dye (PMC+E+). The addition of CoralLoad Concentrate, serving for direct loading of PCR products on agarose gels, is recommended by the manufacturer. When EvaGreen and CoralLoad were present in the PM master mix, flat melting curves were obtained. Due to low raw fluorescence intensities and low steepness at the inflection point, heteroduplex dissociation only slightly contributed to the melting curve, impairing differentiation of multiple heterozygous variants. Most probably, this problem was caused by excitation/emission spectra overlap of EvaGreen dye and the orange/red tracking dyes in CoralLoad. The EpiTect HRM Master Mix led to diminished signal heights at higher dispensation numbers. However, this problem could be solved by upscaling immobilization of biotinylated PCR products to streptavidin coated sepharose beads.

Since different samples do usually not lead to identical absolute fluorescence intensities, all melting curves were normalized between 100% and 0%. In all runs, the melting curve obtained for the triple homozygous variant combination CCC/CCC (wild type) was chosen as reference, and the difference between each curve and the reference curve was plotted against temperature to give a “difference” plot, enhancing visual discrimination. We developed an open source R script for semi-automated fast analysis and visualization for HRM but also for PSQ data.

In order to assess the discrimination power of the 2 SNPs assay and the 3 SNPs assay and the potential of unambiguously identifying variant combinations by coupling HRM to PSQ, we prepared 36 oligodeoxynucleotide controls, covering all possible variant combinations of the three SNPs of interest. The oligodeoxynucleotide controls had to be diluted 1:75,000,000 to obtain Ct values similar to those of biological samples.

In general, the 3 SNPs assay was found to be superior to the 2 SNPs assay. In case of the 2 SNPs assay, one, twelve, and thirteen variant combinations could be unambiguously identified by HRM analysis, PSQ, and HRM coupled to PSQ, respectively. The 3 SNPs assay allowed unambiguous identification of 22, 16, and 34 variant combinations by HRM analysis, PSQ, and by coupling HRM to PSQ, respectively.

Mispriming of the forward primer with the T allele at position rs527559815 resulted in biased but not allele specific PCR. Originally, the 2 SNP assay has not been designed to be allele specific [27], which would require 3′-end mispriming and an additional artificial mismatch site [36]. Due to the PCR bias, a shift in Ct values from triple-homozygous combinations carrying the C allele of rs527559815 to triple-homozygous combinations carrying the respective T allele was observed. This resulted in lower contribution of the DNA strand carrying the T allele at position rs527559815 to both, homo- and heteroduplex melting. In spite of the bias, the triple-heterozygous combination CCC/TTT was the only variant combination that was identified unambiguously by HRM analysis. Particularly, reciprocal heterozygous variants at rs547832288 and rs16906252 could not be distinguished.

With the 3 SNPs assay, 22 out of 36 variant combinations could be identified unambiguously by HRM analysis. While all variant combinations differing at position rs527559815 were distinguishable, this was not the case for twelve out of 24 reciprocal heterozygous variants at rs547832288 and rs16906252. In addition, the melting curves for the reciprocal homozygous variant combinations at rs547832288 and rs16906252, TTC/TTC and TCT/TCT, were identical.

The lower discrimination power for positions rs547832288 and rs16906252 can be explained by similar Tm for reciprocal variants at these positions. We found Tm to be decreased by 0.6 °C and 0.7 °C for triple-homozygous variants when the T allele was at rs547832288 and rs16906252, respectively. A shift of −0.4 °C was obtained for the T allele at rs5275598150. rs547832288, and rs16906252 are in close vicinity in a distance of 18 bp. This region is rich in guanine (G) and cytosine (C). Sequences comprising ten nucleotides up- and downstream of rs547832288 have a GC content of 90% (∆G ≈ −15.0 Kcal/mol) and 70% (∆G ≈ −11.8 Kcal/mol), respectively, those up- and downstream of rs16906252 80% (∆G ≈ −14.8 Kcal/mol) and 60% (∆G ≈ −12.1 Kcal/mol), respectively. rs5275598150 is located 28 bp upstream of rs547832288, surrounded by a region with extremely high GC content (10 nucleotides upstream 100%, ∆G ≈ −15.9 Kcal/mol) and a region with moderate GC content (10 nucleotides downstream 40%, ∆G ≈ −9.4 Kcal/mol). The impact of GC rich regions on HRM analysis is discussed very controversially. According to some studies, HRM data obtained for GC rich regions should be interpreted with caution [19]. Some studies suggest locating GC rich regions at either the 5′ end or the 3′ end of the PCR product [37]. Another strategy is to optimize the PCR-HRM reaction mix by using additives particularly developed for GC rich regions [18]. However, other studies did not find any impact of the GC content on HRM results [38]. When we are designing HRM assays, we generally try to cope with GC rich regions by varying both amplicon length and variant position in the PCR product (in the middle or close to the end). Due to the extended target region of the 3 SNPs assay (23 bp upstream) compared to the 2 SNPs assay, the 3 SNPs assay was found to be superior even for discrimination of rs547832288 and rs16906252. For example, in contrast to the 2 SNPs assay, the 3 SNPs assay enabled discrimination of CCC/CTC and CCC/CCT.

As expected, with increasing number of mispriming sites, the stability of heteroduplexes decreased from double-homozygous to double-heterozygous to triple-heterozygous variants. For the complex melting curve obtained for variant combination CCC/TTT, the three main melting transitions (Tm: 87.56 °C, 85.96 °C, and 83.06 °C) could be assigned to homoduplex melting of CCC/CCC, homoduplex melting of TTT/TTT, and heteroduplex melting, respectively. The two minor transitions causing peak shoulders (Tm: 83.96 °C and 81.86 °C) might result from partial melting of the heteroduplex, because higher decrease in Tm was found for T alleles at positions rs547832288 and rs16906252 (1.3 °C) than for T alleles at positions rs5275598150 and either rs547832288 or rs16906252 (1.0 °C). The occurrence of peak shoulders can, however, not be explained by the closer vicinity of mismatches, because melting curves of variant combinations CCC/TTC and CCC/TCT were identical.

HRM analysis and PSQ are complementary methodologies. HRM analysis using the 3 SNPs assay allowed discrimination between some variant combinations, e.g., TCC/TTT and TTC/TCT, which could not be distinguished by PSQ. On the other hand, PSQ allowed discrimination of six variant combinations, which were not distinguishable by HRM analysis.

In this study, we demonstrated for the first time that by coupling HRM to PSQ, the number of variant combinations that can be identified unambiguously can be substantially increased compared to HRM analysis and PSQ alone. The triple-heterozygous combinations CTC/TCT and CCT/TTC were the only ones that could not be distinguished from each other by the 3 SNPs assay in the coupled approach. Each of the five variant combinations (CCC/CCC, CCC/TCC, CCC/CCT, CCT/CCT, and CCC/CTT) present in the NHGRI Sample Repository could be identified. However, in case of NHGRI sample HG00246, the variant combination determined (CCC/CCT) differs from that given in the Ensembl database (CCC/CCC). Since all our analysis approaches including cluster analysis hint at the variant combination CCC/CCT and the triple-homozygous wildtype variant CCC/CCC was identified in the other samples correctly, we assume that the entry in the Ensembl database is not correct.

By taking both HRM and PSQ data into account, the 3 SNPs assay allowed genotyping of 68 out of 68 cell lines originating mainly from brain, breast, colon, and lung cancer. In addition, the genotype of 50 out of 50 breast tissue samples could be determined. All samples analyzed were found to be homozygous C at rs527559815 and rs547832288, respectively. Two primary glioma cell lines and the non-small cell lung cancer line NCI-H520, but none of the tissue samples, were genotyped homozygous T at rs16906252. The heterozygous genotype of rs16906252 was detected in six cell lines and eight breast tissues.

For some cell lines (GL07, SW480, MDA-MB-453, MCF 10F, and HCT116) atypical melting curves were obtained, lying in between the melting curves obtained for CCC/CCC and CCC/CCT. Sample to sample variability due to differences in sample processing has been reported to impact on genotyping accuracy by HRM analysis [22,39]. However, we did not find the occurrence of atypical melting curves to be associated with specific cell culturing conditions and/or DNA extraction methods. We confirmed our findings by applying a DNase I treatment protocol developed in this study followed by the addition of oligodeoxynucleotide controls.

Manual inspection of the pyrograms obtained for these cell lines and in particular cluster analysis revealed that these cell lines differed in the C/T ratio at position rs16906252 from the regular diploid homozygous CCC/CCC and heterozygous CCC/CCT genotypes. By analyzing mixtures containing the variant combinations CCC/CCC and CCT/CCT in different ratios, the C/T ratio of rs16906252 in these cell lines was determined to be between 1 and 9 by both PSQ and HRM analysis. Thus, alterations in the shape of melting curves, compared to that for CCC/CCT, can be explained by less heteroduplex and more homoduplex formation. Previous studies have already used manual inspection of raw data from pyrograms for quantification of polyploid variants [40,41] or genetic heterogeneity in cancer [42].

Genetic abnormalities have frequently been reported for stable cell lines. For example, the cell line HCT116 has been found to have a gain of part of the q-arm of chromosome 10, resulting in three copies of 10q26 [43,44,45,46]. Cultured cell lines may contain clones of different genotypes and genetic inter-lab differences have also been reported [44]. However, we did not find any differences between HCT116 cultivated in two different laboratories. Genetic abnormalities detected in cell lines may be representative for those found in primary tumors [44,45,47], but they may also be acquired during *in vitro* culturing [48,49,50]. We did not find genetic abnormalities in any of the 24 breast cancer tissue or the 26 breast tissue samples, but in two out of 19 breast cell lines, including one breast cancer cell line and one breast cell line having fibrocystic changes. Although MCF 10A and MCF 10F originate from the same progenitor cell line, we only found copy number gain of the target region in MCF 10F but not in MCF 10A.

DNA from biological samples is frequently limited in amount. We demonstrated that using the 3 SNPs assay, an initial DNA amount of 4 ng and 1 ng (determined fluorometrically) is sufficient to obtain reliable results by HRM analysis and PSQ, respectively.

We could demonstrate that using *k*-means clustering with both HRM and PSQ data is a powerful strategy to overcome the bottleneck of evaluating genotypes manually, e.g., when methods are applied in large cohort studies or clinical routine analysis. The drawback of the software package ScreenClust (Qiagen, Hilden, Germany) is that it does not allow analysis of HRM data from multiple runs [51], hampering appropriate clustering of rare variants. When HRM data from multiple runs were evaluated by cluster analysis, the use of difference curves was most appropriate. Small run-to-run variations, most probably caused by differences in raw fluorescence data, introduced additional noise, impairing accurate clustering using normalized melting curves. These findings are in line with a study using a machine learning approach for cluster analysis, where accurate classification of a single variant from multiple runs required temperature shifting to the mean of a set of plate controls [52]. However, normalized derivative melting curves used in the study of Kanderian et al. do not allow accurate discrimination between very small temperature differences, such as obtained for reciprocal variant combinations from multiple SNPs.

HRM data reduction by PCA prior to cluster analysis, as suggested for dimensionality reduction in some studies [51,53], was not found to be advantageous in this study. Even when PCAs explained almost 100% of the variance, clustering following PCA was less appropriate than direct clustering. Subtle differences between melting curves, obviously crucial for discrimination between multiple variant combinations, were lost by data reduction via PCA.

For PSQ data, we suggest to perform cluster analysis for each variable position separately, particularly when some of the variants are rare. The main advantages of cluster analysis of PSQ data are to simplify representation and evaluation of results and to identify information additionally contained in the pyrograms, such as genetic abnormalities, as detected in this study.

As proof of concept, we demonstrated that HRM analysis coupled with PSQ allows discrimination between 34 of 36 possible combinations of three C>T SNP genotypes in the *MGMT* promoter. The experimental conditions were optimized for the RotorGene, a thermocycler highly suitable for HRM analysis due to its high temperature consistence. It is well known that genotyping of class 3 or 4 SNPs by HRM analysis is more challenging than genotyping of C>T SNPs, belonging to SNP class 1 [17]. However, we expect that HRM analysis coupled with PSQ should allow discrimination between multiple variant combinations of other SNP classes as well, however, this remains to be elucidated. Subjecting one and the same PCR product to HRM analysis and PSQ enables to save time, costs, and DNA amount, compared to performing two separate PCR runs, one for HRM analysis and one for PSQ. We therefore see a potential of PCR-HRM coupled to PSQ to be implemented in routine analysis. In particular, data visualization and *k*-means clustering presented by an open-source R script might contribute to simplify data evaluation in large scale analysis.

## 4. Materials and Methods

Combinations of SNP variants are denoted according to the position of the three SNPs in the *MGMT* promoter (5′ → 3′: rs527559815, rs547832288, rs16906252; Figure 1) as follows. “C” and “T” in front of the slash refer to one DNA strand, “C” and “T” behind the slash to the second DNA strand. For example, “CTC/CCT” refers to a double-heterozygous variant combination, with one DNA strand containing C (rs527559815), T (rs547832288), and C (rs16906252), and the second DNA strand C (rs527559815), C (rs547832288), and T (rs16906252).

### 4.1. Oligodeoxynucleotide Control

Eight oligodeoxynucleotides covering all combinations of the three SNPs in one DNA strand (CCGGATATGCTGGGACAGCCCGCGCCCYTAGAACGCTTT-GCGTCCCGACGCCCGYAGGTCCTCGCGGTGCGYACCGTTTGCGACTTGGT-GAGTGTCT, 97 bp) were purchased (Sigma-Aldrich, Steinheim, Germany) and dissolved in 10 mM Tris-HCl, pH 8.5 buffer (Qiagen, Hilden, Germany). Oligodeoxynucleotides were quantified with the Qubit ssDNA Assay Kit and the Qubit 4 instrument (Thermo Scientific, Vienna, Austria). Oligodeoxynucleotide controls representing the 36 theoretically possible variant combinations for diploid human cells (8 triple-homozygous, 12 double-homozygous, 12 double-heterozygous and 4 triple-heterozygous combinations), were obtained by mixing equal DNA amounts of two oligodeoxynucleotides, respectively (Supplementary Table S1).

### 4.2. Samples with Published Genotypes

DNA extracts of the US National Human Genome Research Institute (NHGRI) Sample Repository for Human Genetic Research were acquired from the Coriell Institute for Medical Research (Camden, NJ, USA). DNA was obtained from lymphoblastoid cell lines (LCLs) established by Epstein-Barr virus transformation of peripheral blood mononuclear cells using the Autopure LS instrument (Qiagen, Hilden, Germany). For samples NA19095 and NA19664, the DNA purification method was not specified. DNA was dissolved in TE buffer (10 mM Tris-HCl, pH 8.0, 1 mM EDTA, pH 8.0) (see the Webpage of the Coriell Institute [54]).

If available, we selected two samples for each genotype of the three SNPs (rs527559815, rs547832288, rs16906252) according to the Ensembl data base entries [55]. Two samples were chosen for CCC/CCC (HG00246, HG01241), CCC/TCC (NA19095, NA19175), CCC/CCT (HG00239, HG00384), and CCT/CCT (HG01784, NA19664). Only one sample was available for CCC/CTT (HG01605).

To investigate effects of polyploidy and allele dilution on genotyping accuracy, the DNA of sample NA19664 (CCT/CCT) was mixed with the DNA of sample HG01241 (CCC/CCC) to obtain different ratios of T allele to C allele (1T:1C (187.5 ng with 187.5 ng), 1T:2C (125 ng with 250 ng), 1T:4C (62.5 ng with 250 ng), 1T:9C (50 ng with 450 ng), and 1T:19C (50 ng with 950 ng).

### 4.3. Cell Lines and Culture

A total of 33 human stable cell lines originating from brain, breast, cervix, colon, lung, peripheral blood, and skin cancer, breast fibrocystic disease or normal breast tissue were included in this study. Stable cell lines were obtained either from the American Type Culture Collection (ATCC; Manassas, VA, USA) or the “Deutsche Sammlung von Mikroorganismen und Zellkulturen” (DSMZ) collection (Braunschweig, Germany) except GLC-4 (kindly donated by Dr. deVries, University of Groningen, The Netherlands) and HMEC (kindly donated by Martha R. Stampfer). In addition, 35 primary human tumor cell lines established from 26 glioma patients as described previously [56] were investigated. Glioma patients were between 15 and 85 years at first surgery (median 55 years) and signed a written informed consent. This study was approved by the local Ethics Commission of the Faculty of Medicine at the Johannes Kepler University Linz (application number E-39-15).

Cell lines were cultured at 37 °C in a humidified 5% CO_2_ incubator and harvested before reaching confluence. Cell pellets were stored at −80 °C until DNA extraction. Details on tissue origin culture media and supplements are provided in Supplementary Table S2.

### 4.4. Tissue Samples

All patients permitted sampling by signing a written informed consent. The study was approved by the Ethics Commission of the Medical University of Vienna (application number EK 2003/260 and EK 2003/366). Tumor breast tissue samples were collected by ultrasound guided needle biopsies from the tumor center of 24 breast cancer patients at diagnosis before receiving radiotherapy, chemotherapy, or hormonal treatment. Breast cancer patients (BRC) were between 32 and 86 years (median 59 years) and none of them had a family history of breast cancer. Normal breast tissue was taken from 26 women without known disease who underwent breast reduction mammoplasty (BRM). The age of these women ranged from 20 to 60 years (median 41 years). Tissue samples were stored in phosphate-buffered saline (PBS, Thermo Scientific, Vienna, Austria) at −80 °C until DNA extraction.

### 4.5. DNA Extraction and Quantification

Genomic DNA was isolated from stable cell lines using the AllPrep DNA/RNA Mini Kit (Qiagen, Hilden, Germany), the High Pure PCR Template Preparation Kit (Roche, Vienna, Austria), QIAamp DNA Mini Kit (Qiagen, Hilden, Germany), or the smart DNA prep (m) kit (Analytik Jena, Jena, Germany) according to manufacturer´s instructions for cell lines. The elution buffers of the AllPrep DNA/RNA Mini Kit and the High Pure PCR Template Preparation Kit contained 10 mM Tris-HCl, pH 8.5, that of the QIAamp DNA Mini Kit 10 mM Tris-HCl, 0.5 mM EDTA, pH 9.0. The smart DNA prep (m) kit contained a Tris-HCl elution buffer without EDTA. MCF7, MDA-MB-231, MDA-MB-468, SK-BR-3, T-47D, ZR-75-1, and HCT116 cell lines were cultivated and extracted under different conditions in different labs (details can be found in Supplementary Table S2).

The QIAamp DNA Mini Kit was used for DNA extraction from tissue samples following the manufacturer’s tissue protocol. Briefly, breast tissue was cut into small pieces and lysed for 4 h before clean-up. Repeated elution with 100 µL elution buffer was performed to increase DNA concentration.

Prior to PCR, isolated DNA and DNA from NHGRI Sample Repository for Human Genetic Research were quantified with the Qubit dsDNA BR Assay Kit or the Qubit dsDNA HS Assay Kit using the Qubit 4 instrument (Thermo Scientific, Vienna, Austria). DNA extracts were stored at −20 °C.

### 4.6. DNase I Treatment

To investigate the influence of residues from the DNA isolation process on genotyping accuracy, 17 DNA extracts from cell lines (HeLa, HT-29, BT-20, HCC1937, HMEC, Hs 578T, MCF7 (grown in DMEM media), MDA-MB-231 (grown in L-15 media or RPMI-1640), A431, GL01, GL06, GL07, GL08c, GL11, GL19e, and GL23b) were used.

DNA extracts were diluted to 5 ng/µL with RNase-free water prior to digestion in order to obtain the same concentration of residues in DNase I treated extracts and extracts from biological samples subjected to PCR. 3.4 U RNase-free DNase I (Qiagen, Hilden, Germany) was added to DNA extracts with a DNA amount ≤ 340 ng and reaction took place in the iCycler instrument (Bio-Rad, Vienna, Austria) as follows: incubation for 20 min at 37 °C, enzyme inactivation for 10 min at 75 °C, and cooling phase for 1 min to 25 °C. To ensure complete inactivation of DNase I, samples were additionally vortexed for 30 s. After centrifugation for 10 min at 18,400× *g*, the supernatant was taken and DNase I treatment was repeated. The DNA concentration was found to be below the LOD of the Qubit dsDNA HS Assay Kit (Thermo Scientific, Vienna, Austria), indicating that DNase I treatment was successful. In order to test for remaining DNase activity, untreated DNA extract was added to an aliquot of the 2× DNase I treated extracts and incubated 1 h at 37 °C. We did not find a difference between the DNA concentration before and after incubation, indicating lack of DNase activity. In addition, all DNase I treated samples were checked for absence of PCR product. DNase I treated samples were stored at −20 °C until PCR.

### 4.7. PCR and HRM Acquisition

The nucleotide sequence of the *MGMT* promoter (Genbank GRCh38.p13 chr 10 NC_000010.11: range 129466653 to 129467477) was retrieved from the National Center for Biotechnology Information (NCBI) data base.

An HRM primer set for genotyping SNP rs16906252 was taken from literature [27]. In order to be applicable for PSQ, the reverse primer was biotinylated and a sequencing primer was designed using the PyroMark Assay Design Software 2.0.1.15 (Qiagen, Hilden, Germany) considering the requirements for primer design for both HRM and PSQ (Figure 1). In addition, a novel forward primer and a sequencing primer were designed to cover the three SNPs of interest (3 SNPs assay). Primer sets for the 2 SNPs and 3 SNPs assay (Figure 1) were purchased from Sigma-Aldrich (Steinheim, Germany) or Eurofins (Ebersberg, Germany). Primer sequences are listed in Table 1.

PCR was performed in 25 µL reaction volumes in the Rotor-Gene Q instrument with 72-well rotor (Qiagen, Hilden, Germany) using 200 nM of each primer. For biological samples, 10 ng DNA (2 µL of 5 ng/µL) was used. A no template control (2 µL nuclease-free H_2_O) was included in each run. To achieve Ct values similar to the Ct values obtained for biological samples, 10 ng of oligodeoxynucleotide controls were diluted 1:75,000,000 in 10 mM Tris-HCl, pH 8.5 buffer (Qiagen, Hilden, Germany).

For the investigation of the influence of residues from the DNA isolation process on genotyping accuracy, 2 µL of 2× DNase I treated samples were used with 2 µL of 1:75,000,000 diluted oligodeoxynucleotide controls, latter replacing nuclease-free H_2_O in the reaction mix. In addition, the minimal DNA input required for accurate genotyping was investigated in a range from 10 ng to 0.01 ng. All samples were analyzed in at least two independent PCR-HRM runs including two PCR replicates.

The annealing temperature was optimized and the performance of the following PCR reaction mixes compared: 1× PyroMark PCR Master Mix from the PyroMark PCR Kit (Qiagen, Hilden, Germany) with and without 1× CoralLoad Concentrate (Qiagen, Hilden, Germany) and with and without 1× EvaGreen dye (Biotium, Fremont, CA, USA), and 1× EpiTect HRM Master Mix from the EpiTect HRM PCR Kit (Qiagen, Hilden, Germany). Amplification was performed with initial activation at 95 °C for 15 min (PyroMark PCR Master Mix) or 5 min (EpiTect HRM Master Mix), followed by 50 cycles of denaturation at 94 °C for 10 s, annealing at 60 °C for 20 s, and elongation at 72 °C for 20 s and final elongation at 72 °C for 10 min. To allow heteroduplex formation, PCR products were denatured at 95 °C for 1 min and hybridized at 40 °C for 1 min. HRM acquisition followed immediately with a ramp from 65 °C to 95 °C with 0.1 °C/hold (2 s) and gain optimization (70% before melt).

Quality and yield of PCR products were assessed by gel electrophoresis (2% agarose (Sigma-Aldrich, Vienna, Austria) gel containing 1× GelRed (Biotium, Fremont, CA, USA), 1× TAE buffer (Sigma-Aldrich, Vienna, Austria) and UV-detection.

### 4.8. PSQ of PCR-HRM Products

PSQ was performed using the PyroMark Q24 Vacuum Workstation and PyroMark Q24 Advanced instrument with PyroMark Q24 Advanced CpG Reagents and PyroMark Q24 Advanced Accessories (all Qiagen, Hilden, Germany) according to the manufacturer’s instructions. Primer sequences, sequences to analyze, and dispensation orders are listed in Table 1. DNA immobilization was optimized in a range from 5–39 µL biotinylated PCR-HRM product (pool of technical replicates) with 1 µL Streptavidin Sepharose High Perfomance beads (GE Healthcare, Germany) and 40 μL PyroMark Binding Buffer per 80 µL immobilization reaction. 22.5 µL biotinylated PCR-HRM product, 1.5 µL Streptavidin Sepharose High Performance beads, and 60 μL PyroMark Binding Buffer per 120 µL immobilization reaction was also tested. DNA immobilization was performed under agitation for 10 min at 1400 rpm. The captured PCR product was denatured, washed, and finally the biotinylated strand was transferred into a PyroMark Q24 Plate containing 20 µL of 0.375 μM sequencing primer in PyroMark Annealing Buffer. The plate was heated at 80 °C for 5 min and then transferred into the instrument holding the PyroMark Q24 Cartridge loaded according to pre-run information provided by the PyroMark Q24 Advanced software 3.0.0 (Qiagen, Hilden, Germany).

### 4.9. Data Analysis

Amplification and melting curves obtained by PCR-HRM were assessed using the Rotor-Gene Q Series Software 2.3.1 (Qiagen, Hilden, Germany). Normalization intervals before and after the major fluorescence decrease were 78.57–80.07 °C and 87.74–89.24 °C (2 SNPs assay) and 79.86–81.36 °C and 90.24–91.74 °C (3 SNPs assay). Normalized data obtained by “line of best fit” and light convolution by “sliding window of points” algorithms were exported. PSQ data were evaluated with the PyroMark Q24 Advanced software 3.0.0 (Qiagen, Hilden, Germany) using default settings, and “Peak Height Report” and “SNP Analysis Results Report” were exported.

Exported data were analyzed and presented graphically using R version 3.6.2 [57]. R packages and commands used can be found in Supplementary Files R scripts 1–3.

Derivative melting curves were calculated from the normalized melting curves by applying Savitzky-Golay filtering for third-degree polynomials. In case of peaks, the approximate melting temperature (Tm) was assessed from the peak maximum. For peak shoulders, the second derivative was used.

To obtain difference curves, for each temperature the mean of the normalized fluorescence of the melting curve obtained for the respective wildtype control was subtracted from the normalized fluorescence of each technical replicate.

Peak heights obtained from PSQ were analyzed by non-parametric Kruskal-Wallis rank sum test followed by post-hoc Dunn’s test with Holm’s *p*-value adjustment. An adjusted *p*-value ≤ 0.05 was considered as statistically significant. Since peak heights for A are higher than those obtained for the other deoxynucleotides, they were adjusted by a factor of 0.9.

Cluster analysis was performed for data obtained with the 3 SNPs assay. Peak heights at variable positions were normalized and given as ratios. For normalization, we used peak heights obtained for dispensations 2 (G), 4 (G), 5 (C), 7 (G), 11 (A), and 12 (G), each of them resulting in the incorporation of a single nucleotide. Data resulting in a normalized peak ratio higher 0.15 at dispensation 6 (0T) were excluded. For identification of the appropriate cluster model, *k*-means clustering was applied on data from normalized melting curves, difference curves, and normalized peak heights at variable positions. Data obtained by PCR-HRM were also analyzed by principal component analysis (PCA) prior to *k*-means clustering. Number of clusters was determined by scree plot and careful inspection of data. Number of PCs was determined by scree plot, eigenvalues >1 and the requirement that PCs should explain at least 90% of the variance.

## Figures and Tables

**Table 1:** Primer sequences and PCR-HRM-PSQ assay characteristics.

Assay	Primer Sequence (5′–3′)	Sequence to Analyze/Dispensation Order (5′–3′)	SNPs Targeted
2 SNPs assay ^1^	fw: GCCCCTAGAACGCTTTGCGTC [27]	GCGTCCCGAC- GCCCGYAGGTCCTCGCGGTGCGYACCG/	rs547832288
(74 bp)	rev: AGACACTCACCAAGTCGCAAACG [27]	CGCGTCGACGCAGCTAGTCTCGCGTGCAGCTACG	rs16906252
	rev: [btn]-AGACACTCACCAAGTCGCAAACG		
	seq: CCCATAGAACGCTTT		
3 SNPs assay	fw: CCGGATATGCTGGGACAGCC	GCCCGCGCCCYTAGAACGCTTTGCGTCCCGAC- GCCCGYAGGTCCTCGCGGTGCGYACCG/	rs527559815
(97 bp)	rev: [btn]-AGACACTCACCAAGTCGCAAACG	CGCGCTGCCTAGACGCTGCGTCGACGCAGCTAG- TCTCGCGTGCAGCTACG	rs547832288
	seq: GGATATGCTGGGACA		rs16906252

^1^ The nucleotide in the forward primer marked in bold is the major allele of rs527559815. The sequencing primer has a mispriming site at rs527559815S1. Reverse primers are labeled with biotin (btn).

## Data Availability

The datasets generated during the current study are available from the corresponding author on reasonable request.

## Supplementary Materials

The following are available online at www.mdpi.com/article/10.3390/ijms222212527/s1.
