# Supplementary material for: Discrimination between 34 of 36 Possible Combinations of Three C>T SNP Genotypes in the MGMT Promoter by High Resolution Melting Analysis Coupled with Pyrosequencing Using A Single Primer Set
Source: Int J Mol Sci. 2021 Nov 20;22(22):12527. doi: 10.3390/ijms222212527 (PMC8621402; doi:10.3390/ijms222212527)
Supplement: Supplementary file 1 [file ijms-22-12527-s001.zip › Supplementary_Figures_MGMT_SNP.pdf]

International Journal of *Molecular Sciences*

MDPI

Supplementary Figures

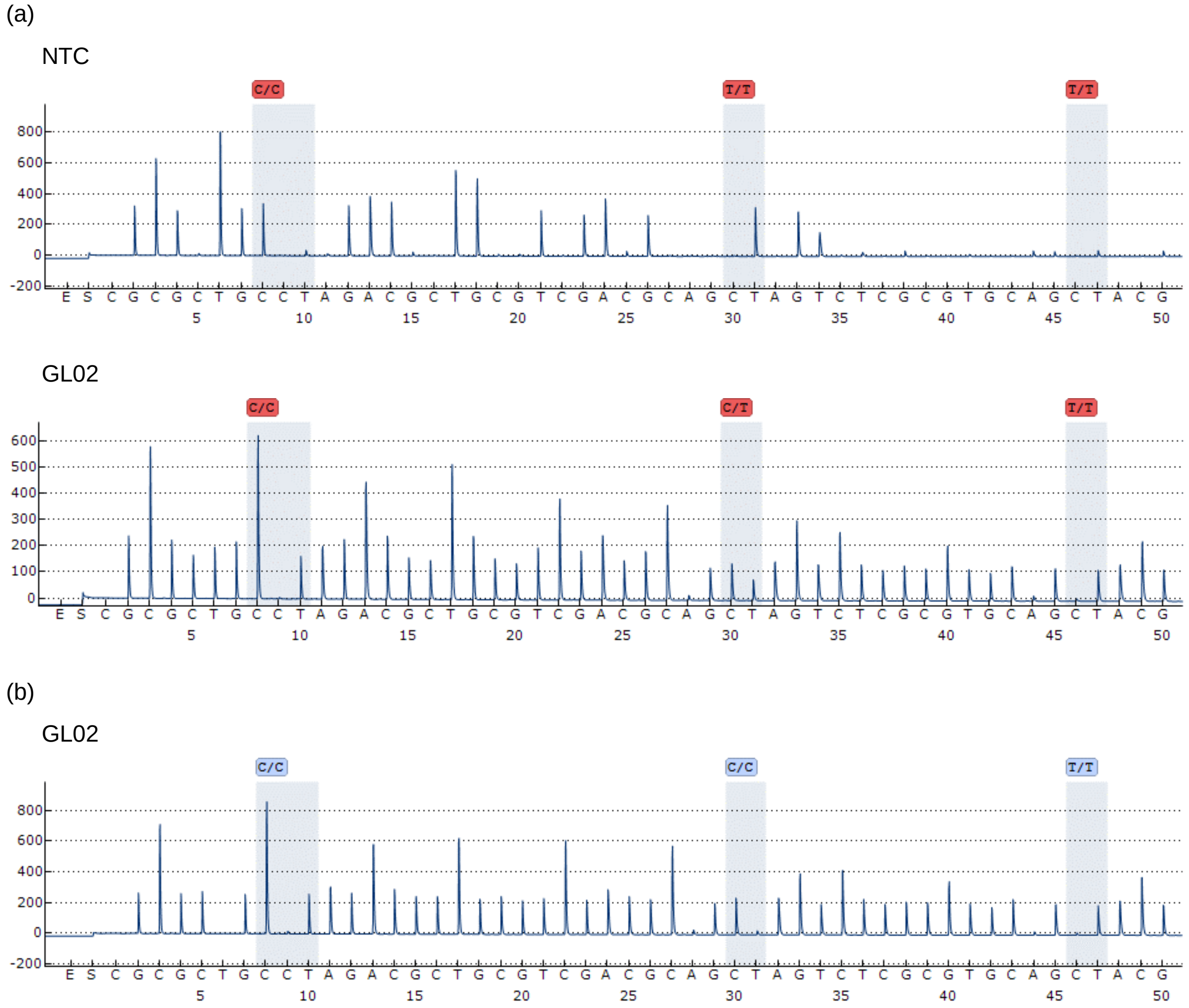

Figure S1. Pyrograms of PCR products where (**a**) primer dimers occurred in an NTC and in sample GL02 and (**b**) sample GL02 without primer dimers. The primer dimers could be identified by the presence of a peak at dispensation 6 (0T).

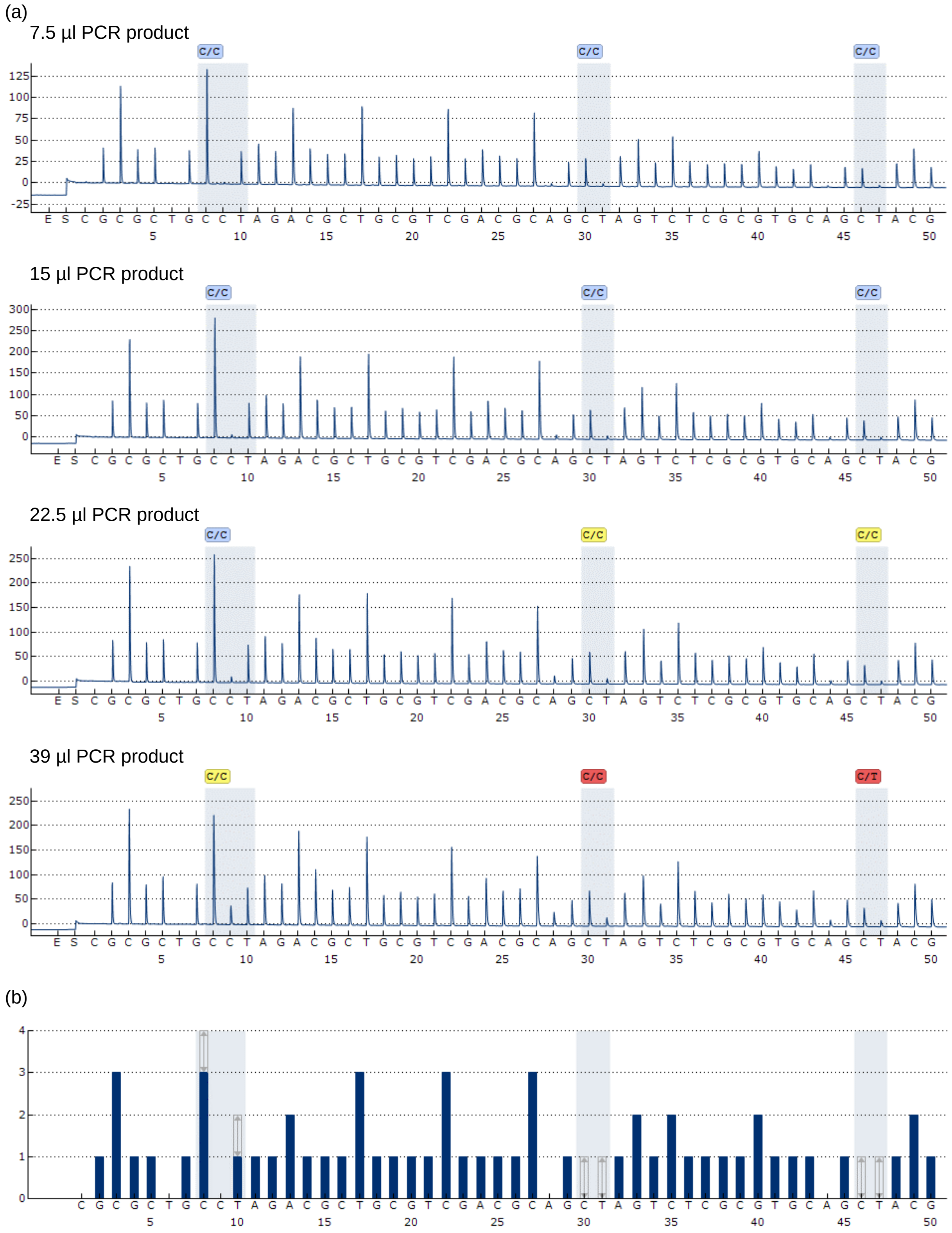

Figure S2. Varied amounts of PCR product (mix ET) (**a**) 7.5 µl–39 µl when using 1 µl streptavidin beads in a total volume of 80 µl for immobilization reaction. (**b**) The histogram visualizing the dispensation order and the fold nucleotide incorporation according to the DNA sequence. Representative pyrograms were shown for sample HT-29 using the 3 SNPs assay assessing the variable positions (shaded in blue-gray) for rs527559815 (NC_000010.11: g.129467237C>T), rs547832288 (NC_000010.11: g.129467264C>T) and rs16906252 (NC_000010.11: g.129467281C>T). Warning notes (yellow: uncertain and red: failed analysis results) and/or low peak heights for dispensations 41–43, 45 48, and 50 were obtained impairing accurate genotyping of rs16906252.

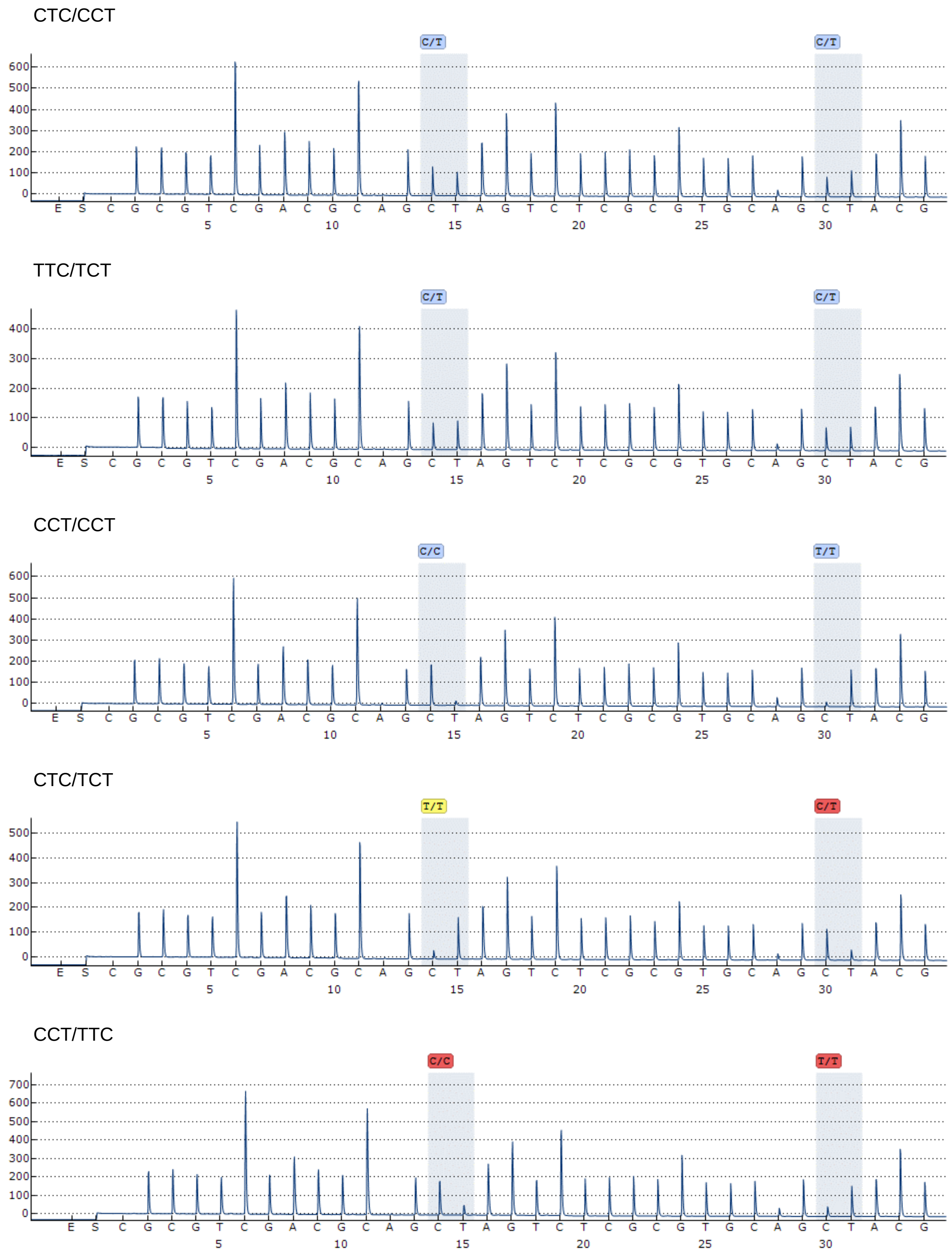

Figure S3. Biased amplification of the C allele of rs527559815 allowed discrimination of double- and triple-heterozygous variant combinations, which were heterozygous for rs527559815. This led to altered C/T ratios in the positions rs547832288 and rs16906252 and warning notes (yellow: uncertain and red: failed analysis results) when both variants of rs527559815 were present in CTC/TCT and CCT/TTC compared to CTC/CCT, TTC/TCT, and CCT/CCT, where rs527559815 was homozygous.

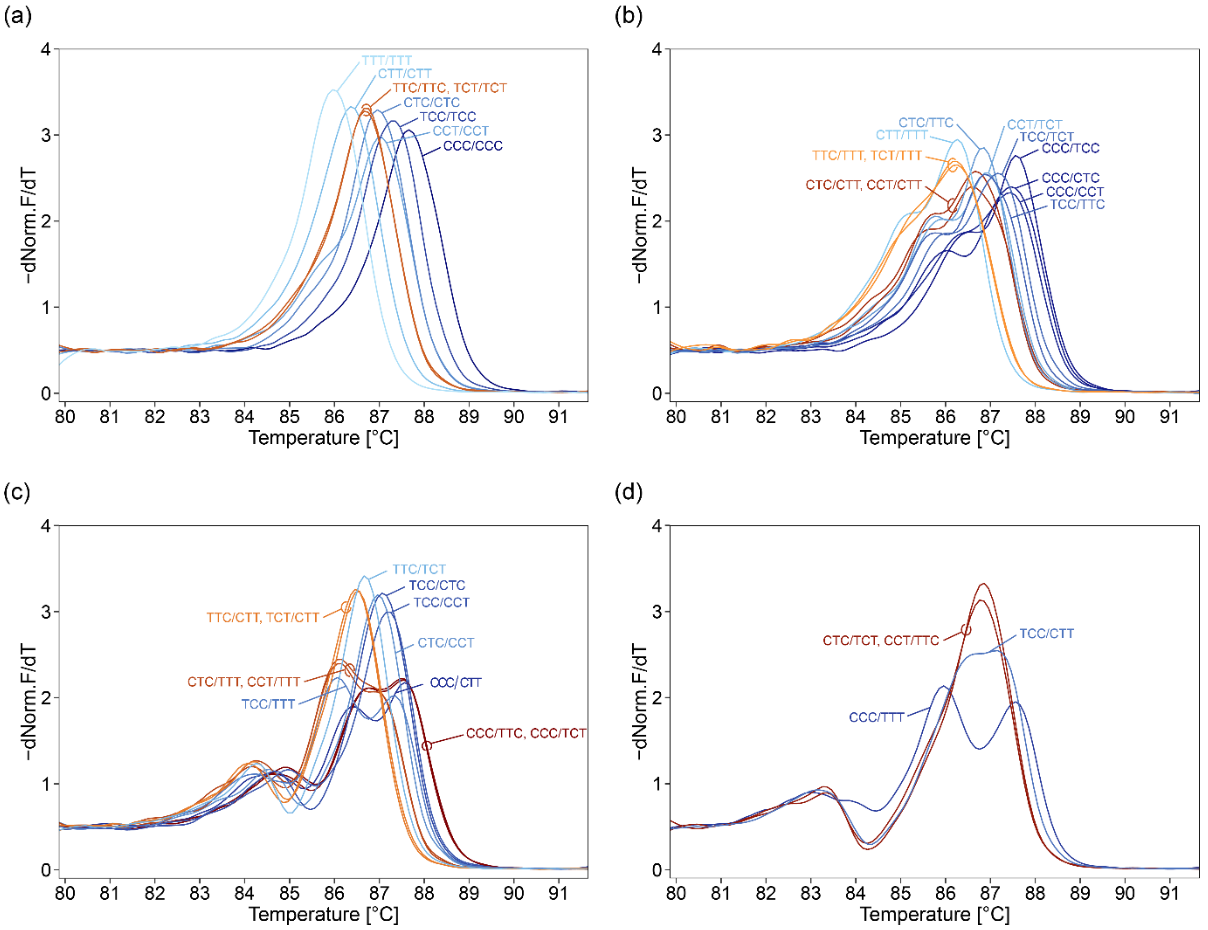

Figure S4. Derivative melting curves of oligonucleotide controls obtained with the 3 SNPs assay for (**a**) triple-homozygous, (**b**) double-homozygous, (**c**) double-heterozygous, and (**d**) triple-heterozygous variant combinations. Distinguishable melting curves were shown in blue and identical melting curves in orange/red. One technical replicate instead of the mean were shown to maintain curve shapes.

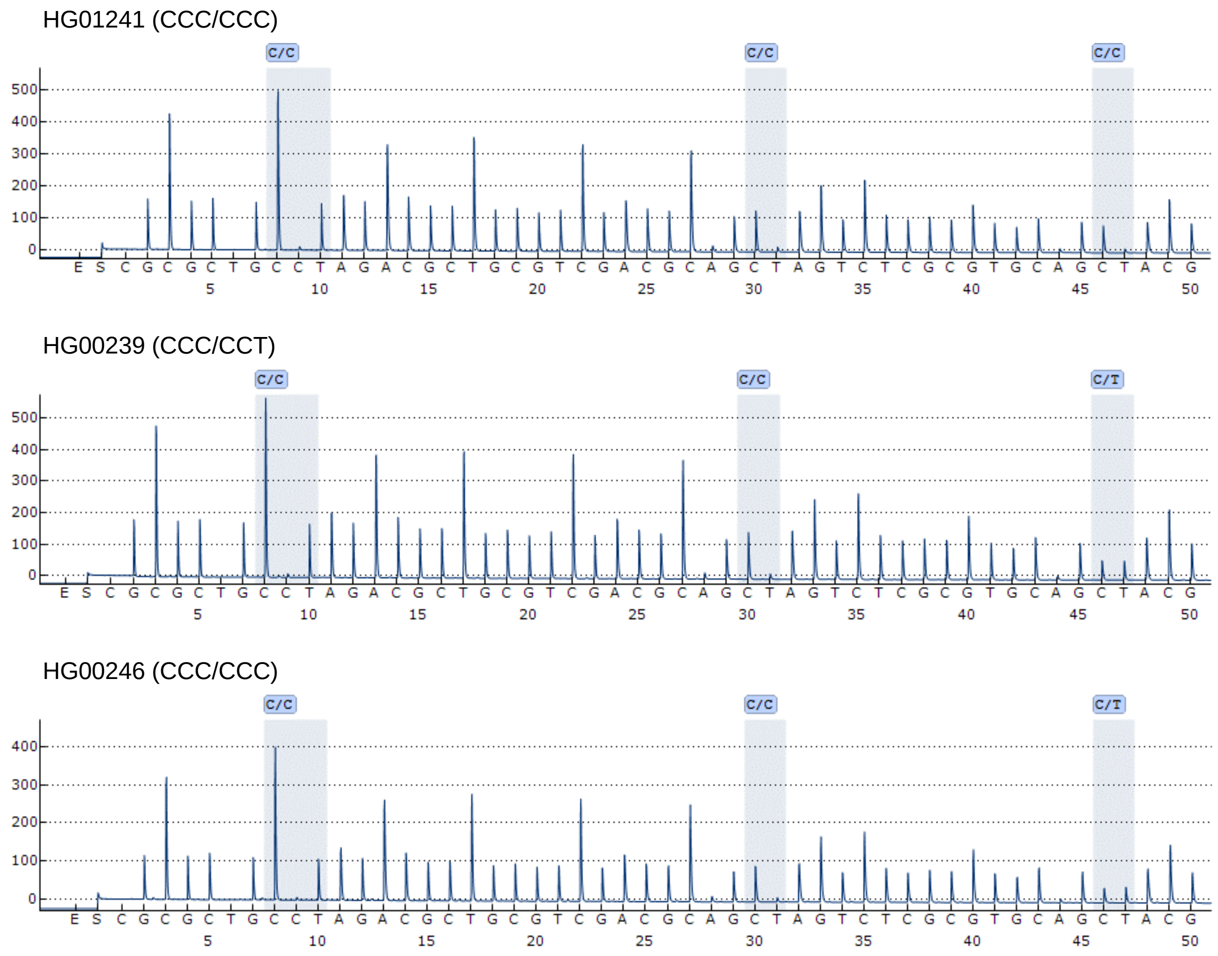

Figure S5. Pyrogramms obtained for NHGRI samples HG01241, HG00239, and HG00246 using the 3 SNPs assay. Genotypes found for HG01241 (CCC/CCC) and HG00239 (CCC/CCT) were in line with those given in the Ensembl data base, while HG00246 (CCC/CCC) was genotyped CCC/CCT.

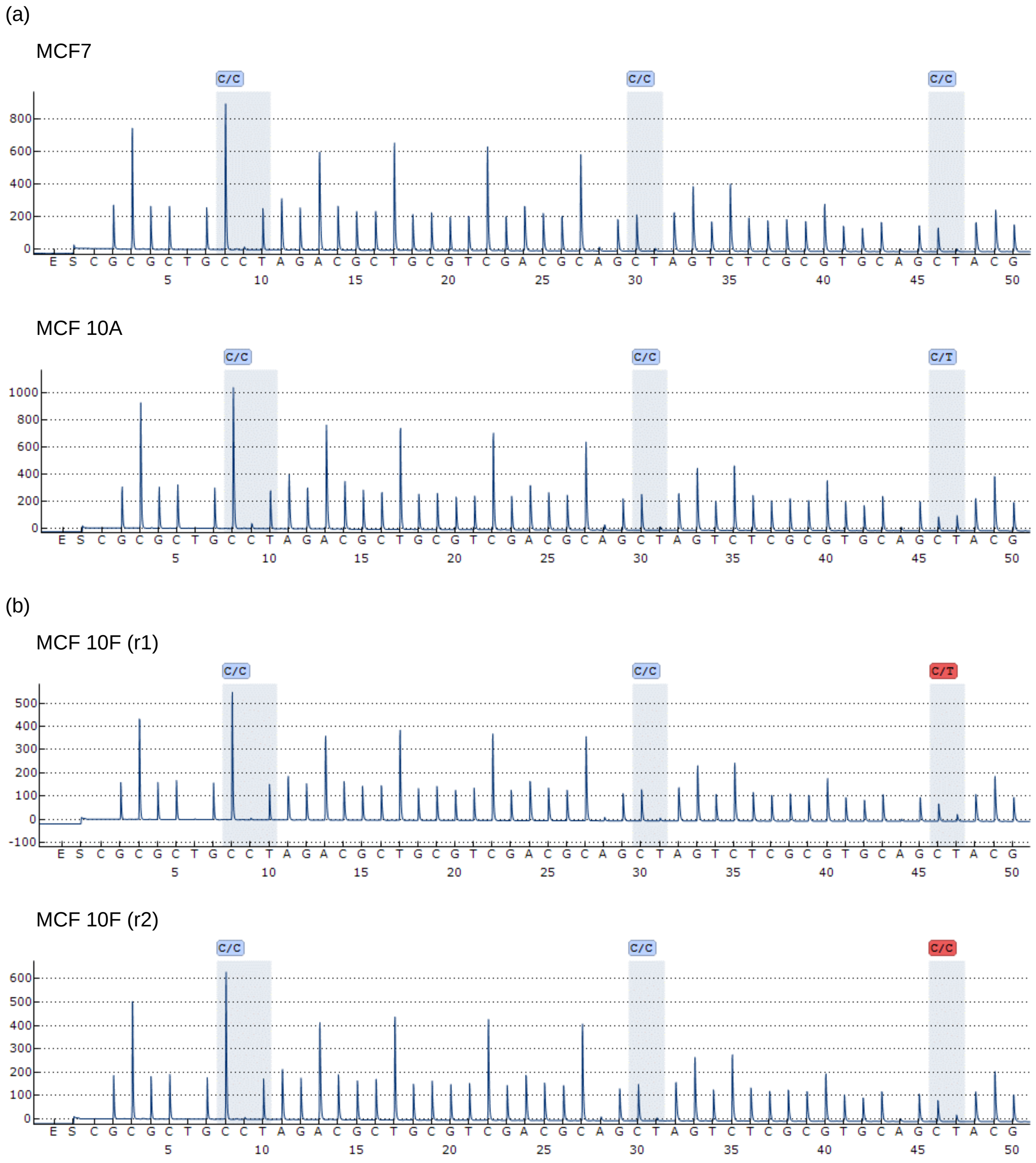

Figure S6. (**a**) Regular and (**b**) irregular pyrograms obtained for stable cell lines. MCF7 was genotyped CCC/CCC and MCF 10A was genotyped CCC/CCT without warning messages, while for technical replicates (r1, r2) for MCF 10F either CCC/CCT or CCC/CCC was obtained. Peaks for both the C and to a lower extent for T allele of rs16906252 were present, resulting in the "failed genotype determination" warning message (red). In contrast, no T allele was found in the pyrogram of MCF7.

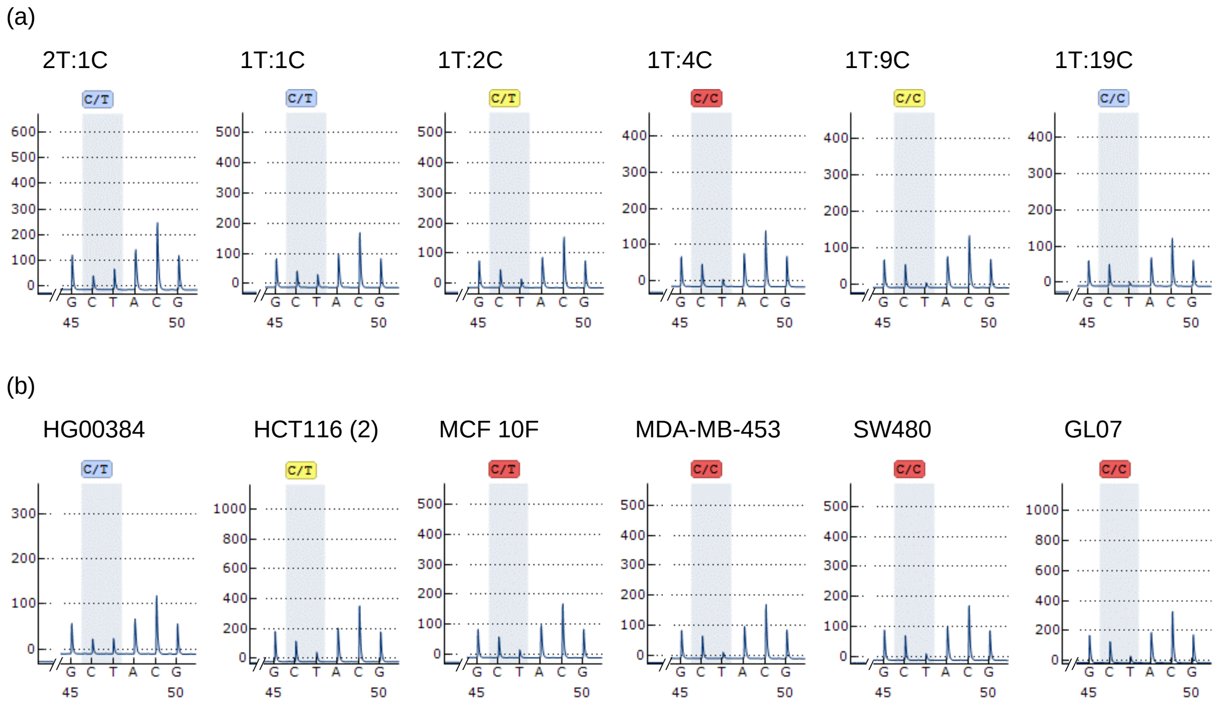

Figure S7. Part of the pyrograms (3 SNPs assay) showing the variable position for rs16906252. (**a**) Dilution of the CCT allele in CCC in ratios for rs16906252 of 2T:1C–1T:19C. (**b**) NHGRI sample HG00384 (CCC/CCT) and cell lines for which atypical melting curves were obtained. Warning notes were yellow for uncertain and red for failed analysis results. Regular pyrograms were only obtained for 1T:1C and HG00384. Increase in the C/T signal heights ratio could be observed by higher dilution of the CCT allele.

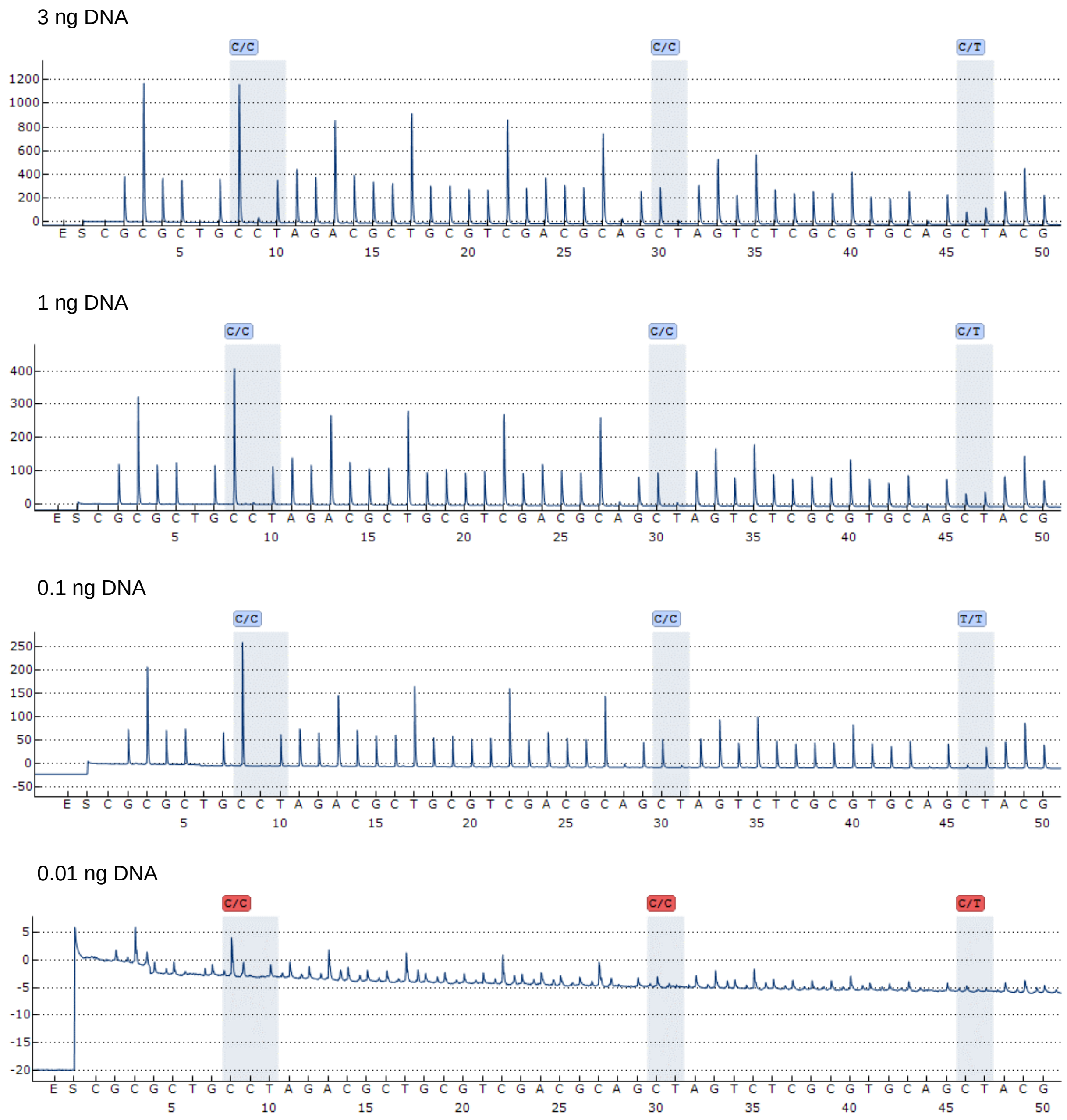

Figure S8. Pyrograms for obtained after PCR with reduced initial DNA amount of sample BRM13. Accurate genotyping for the variant combination CCC/CCT was possible down to 1 ng initial DNA. With a DNA amount of 0.1 ng, only one allele (CCT) was amplified, while no PCR product was obtained when 0.01 ng DNA was used for PCR.
