# Supplementary material for: Discrimination between 34 of 36 Possible Combinations of Three C>T SNP Genotypes in the MGMT Promoter by High Resolution Melting Analysis Coupled with Pyrosequencing Using A Single Primer Set
Source: Int J Mol Sci. 2021 Nov 20;22(22):12527. doi: 10.3390/ijms222212527 (PMC8621402; doi:10.3390/ijms222212527)
Supplement: Supplementary file 1 [file ijms-22-12527-s001.zip › Supplementary_Tables_MGMT_SNP.pdf]

International Journal of
Molecular Sciences

MDPI

Supplementary Tables

Table S1. Characteristics of the oligodeoxynucleotide controls representing the 36 possible diploid variant combinations of the three C>T SNPs rs527559815, rs547832288 and rs16906252.

Variant Combination	Strand 1	Strand 2	Genetic State
CCC/CCC	CCC	CCC	triple-homozygous
CCC/TCC	CCC	TCC	double-homozygous
CCC/CTC	CCC	CTC	double-homozygous
CCC/CCT	CCC	CCT	double-homozygous
CCC/TTC	CCC	TTC	double-heterozygous
CCC/TCT	CCC	TCT	double-heterozygous
CCC/CTT	CCC	CTT	double-heterozygous
CCC/TTT	CCC	TTT	triple-heterozygous
TCC/TCC	TCC	TCC	triple-homozygous
TCC/CTC	TCC	CTC	double-heterozygous
TCC/CCT	TCC	CCT	double-heterozygous
TCC/TTC	TCC	TTC	double-homozygous
TCC/TCT	TCC	TCT	double-homozygous
TCC/CTT	TCC	CTT	triple-heterozygous
TCC/TTT	TCC	TTT	double-heterozygous
CTC/CTC	CTC	CTC	triple-homozygous
CTC/CCT	CTC	CCT	double-heterozygous
CTC/TTC	CTC	TTC	double-homozygous
CTC/TCT	CTC	TCT	triple-heterozygous
CTC/CTT	CTC	CTT	double-homozygous
CTC/TTT	CTC	TTT	double-heterozygous
CCT/CCT	CCT	CCT	triple-homozygous
CCT/TTC	CCT	TTC	triple-heterozygous
CCT/TCT	CCT	TCT	double-homozygous
CCT/CTT	CCT	CTT	double-homozygous
CCT/TTT	CCT	TTT	double-heterozygous
TTC/TTC	TTC	TTC	triple-homozygous
TTC/TCT	TTC	TCT	double-heterozygous
TTC/CTT	TTC	CTT	double-heterozygous
TTC/TTT	TTC	TTT	double-homozygous
TCT/TCT	TCT	TCT	triple-homozygous
TCT/CTT	TCT	CTT	double-heterozygous
TCT/TTT	TCT	TTT	double-homozygous
CTT/CTT	CTT	CTT	triple-homozygous
CTT/TTT	CTT	TTT	double-homozygous
TTT/TTT	TTT	TTT	triple-homozygous

Table S2. Samples characteristics and genotyping results (3 SNPs assay) for stable cell lines (T98G – A431) and primary glioma cell lines (GL01 – GL26).

Cell Line	Tissue Origin	Culture Condition	Extraction Kit	HRM Analysis	*k*3 (HRM)	PSQ results	*k*3 (rs16906252)	*k*4 (rs16906252)
T98G	brain, GBM	RPMI-1640, 7% FCS	QiaAmp	CCC/CCC	cluster a	n.a.	-	-
U251	brain, GBM	MEM, 10% FCS, N,P	AllPrep	CCC/CCC	cluster a	CCC/CCC	cluster 1	cluster 1
AU565†	breast cancer	RPMI-1640, 10% FCS, 1% P/S	HighPure	CCC/CCC	cluster a	n.a.	-	-
BT-20	breast cancer	MEM, 10% FCS, N,P	AllPrep	CCC/CCC	cluster a	CCC/CCC	cluster 1	cluster 1
BT-474	breast cancer	medium 1, 10% FCS, 1% P/S	HighPure	CCC/CCC	cluster a	n.a.	-	-
BT-549	breast cancer	RPMI-1640, 5% FCS, 1% P/S	smartDNA	CCC/CCC	cluster a	CCC/CCC	cluster 1	cluster 1
CAL-51	breast cancer	RPMI-1640, 5% FCS, 1% P/S	HighPure	CCC/CCC	cluster a	CCC/CCC	cluster 1	cluster 1
CAMA-1	breast cancer	RPMI-1640, 5% FCS, 1% P/S	HighPure	CCC/CCC	cluster a	n.a.	-	-
HCC1143	breast cancer	RPMI-1640, 5% FCS, 1% P/S	HighPure	CCC/CCC	cluster a	CCC/CCC	cluster 1	cluster 1
HCC1937	breast cancer	RPMI-1640, 5% FCS, 1% P/S	HighPure	CCC/CCC	cluster a	CCC/CCC	cluster 1	cluster 1
HMEC‡	breast	MEGM, 1% P/S	HighPure	CCC/CCC	cluster a	CCC/CCC	cluster 1	cluster 1
Hs 578T	breast cancer	DMEM/high, 10% FCS, 1% P/S, I	HighPure	CCC/CCC	cluster a	CCC/CCC	cluster 1	cluster 1
MCF7 (1)	breast cancer	DMEM/high, 10% FCS	AllPrep	CCC/CCC	cluster a	CCC/CCC	cluster 1	cluster 1
MCF7 (2)		RPMI-1640, 5% FCS, 1% P/S	HighPure	CCC/CCC	cluster a	n.a.	-	-
MCF 10A§	breast, FCC	MEGM, 1% P/S, C	smartDNA	CCC/CCT	cluster b	CCC/CCT	cluster 2	cluster 2
MCF 10F§	breast, FCC	medium 2, 5% HS, 1% P/S, C,E,H,I,	HighPure	atypical	cluster c	CC?/CC?	cluster 1	cluster 4
MDA-MB-231 (1)	breast cancer	L-15 Leibovitz, 10% FCS, G	AllPrep	CCC/CCC	cluster a	CCC/CCC	cluster 1	cluster 1
MDA-MB-231 (2)		RPMI-1640, 5% FCS, 1% P/S	HighPure	CCC/CCC	cluster a	CCC/CCC	cluster 1	cluster 1
MDA-MB-453	breast cancer	RPMI-1640, 10% FCS, 1% P/S	HighPure	atypical	cluster c	CC?/CC?	cluster 1	cluster 4
MDA-MB-468 (1)	breast cancer	MEM, 10% FCS	AllPrep	CCC/CCC	cluster a	CCC/CCC	cluster 1	cluster 1
MDA-MB-468 (2)		RPMI-1640, 5% FCS, 1% P/S	smartDNA	CCC/CCC	cluster a	CCC/CCC	cluster 1	cluster 1
SK-BR-3† (1)	breast cancer	DMEM/high, 10% FCS	AllPrep	CCC/CCC	cluster a	n.a.	-	-
SK-BR-3† (2)		RPMI-1640, 5% FCS, 1% P/S	HighPure	CCC/CCC	cluster a	n.a.	-	-
T-47D (1)	breast cancer	DMEM/high, 10% FCS	AllPrep	CCC/CCC	cluster a	n.a.	-	-
T-47D (2)		RPMI-1640, 5% FCS, 1% P/S	HighPure	CCC/CCC	cluster a	n.a.	-	-
ZR-75-1 (1)	breast cancer	RPMI-1640, 10% FCS	AllPrep	CCC/CCC	cluster a	n.a.	-	-
ZR-75-1 (2)		RPMI-1640, 5% FCS, 1% P/S	HighPure	CCC/CCC	cluster a	n.a.	-	-

Cell Line	Tissue Origin	Culture Condition	Extraction Kit	HRM Analysis	*k*3 (HRM)	PSQ results	*k*3 (rs16906252)	*k*4 (rs16906252)
HCT116 (1)	colon cancer	McCoy´s 5A, HEPES, 10% FCS, G	AllPrep	atypical	cluster c	CC?/CC?	cluster 1,2	cluster 4
HCT116 (2)		McCoy´s 5A, HEPES, 10% FCS, 1% P/S	AllPrep	atypical	cluster c	CC?/CC?	cluster 1	cluster 4
HT-29	colon cancer	DMEM/high, 10% FCS, 1% P/S	QiaAmp	CCC/CCC	cluster a	CCC/CCC	cluster 1	cluster 1
SW480	colon cancer	MEM, 10% FCS	AllPrep	atypical	cluster c	CC?/CC?	cluster 1	cluster 4
A549	lung cancer	RPMI-1640, 10% FCS	QiaAmp	CCC/CCC	cluster a	n.a.	-	-
DMS 114	lung cancer	RPMI-1640, 10% FCS	QiaAmp	CCC/CCC	cluster a	n.a.	-	-
GLC-4	lung cancer	RPMI-1640, 10% FCS	QiaAmp	CCC/CCC	cluster a	CCC/CCC	cluster 1	cluster 1
HCC827	lung cancer	RPMI-1640, 10% FCS	QiaAmp	CCC/CCC	cluster a	n.a.	-	-
NCI-H520	lung cancer	RPMI-1640, 10% FCS	QiaAmp	CCT/CCT	cluster b	CCT/CCT	cluster 3	cluster 3
NCI-H1703	lung cancer	RPMI-1640, 10% FCS	QiaAmp	CCC/CCC	cluster a	n.a.	-	-
SW 1573	lung cancer	DMEM/high, 10% FCS	QiaAmp	CCC/CCC	cluster a	CCC/CCC	cluster 1	cluster 1
HeLa	cervix cancer	RPMI-1640, 5% FCS, 1% P/S	HighPure	CCC/CCC	cluster a	CCC/CCC	cluster 1	cluster 1
A431	skin cancer	MEM, 10% FCS, 1% P/S	QiaAmp	CCC/CCC	cluster a	CCC/CCC	cluster 1	cluster 1
GL01	brain, GBM	RPMI-1640, 7% FCS	QiaAmp	CCC/CCC	cluster a	CCC/CCC	cluster 1	cluster 1
GL02	brain, GS	RPMI-1640, 7% FCS	QiaAmp	CCT/CCT	cluster b	CCT/CCT	cluster 3	cluster 3
GL03	brain, GBM	RPMI-1640, 7% FCS	QiaAmp	CCC/CCC	cluster a	n.a.	-	-
GL04	brain, GBM	RPMI-1640, 7% FCS	QiaAmp	CCC/CCC	cluster a	n.a.	-	-
GL05	brain, GBM	RPMI-1640, 7% FCS	QiaAmp	CCC/CCC	cluster a	n.a.	-	-
GL06	brain, GBM	RPMI-1640, 7% FCS	QiaAmp	CCC/CCC	cluster a	CCC/CCC	cluster 1	cluster 1
GL07	brain, GBM	RPMI-1640, 7% FCS	QiaAmp	atypical	cluster c	CC?/CC?	cluster 1	cluster 4
GL08a	brain, GBM	RPMI-1640, 7% FCS	QiaAmp	CCC/CCC	cluster a	n.a.	-	-
GL08b	brain, rec. GBM	RPMI-1640, 7% FCS	QiaAmp	CCC/CCC	cluster a	n.a.	-	-
GL08c	brain, rec. GBM	RPMI-1640, 7% FCS	QiaAmp	CCC/CCC	cluster a	CCC/CCC	cluster 1	cluster 1
GL09	brain, GBM	RPMI-1640, 7% FCS	QiaAmp	CCC/CCC	cluster a	n.a.	-	-
GL10	brain, GBM	RPMI-1640, 7% FCS	QiaAmp	CCC/CCC	cluster a	n.a.	-	-
GL11	brain, GBM	RPMI-1640, 7% FCS	QiaAmp	CCC/CCC	cluster a	CCC/CCC	cluster 1	cluster 1
GL12	brain, GBM	RPMI-1640, 7% FCS	QiaAmp	CCC/CCC	cluster a	n.a.	-	-
GL13	brain, GBM	RPMI-1640, 7% FCS	QiaAmp	CCC/CCC	cluster a	n.a.	-	-
GL14	brain, GBM	RPMI-1640, 7% FCS	QiaAmp	CCC/CCC	cluster a	n.a.	-	-
GL15	brain, GBM	RPMI-1640, 7% FCS	QiaAmp	CCC/CCC	cluster a	CCC/CCC	cluster 1	cluster 1

Cell Line	Tissue Origin	Culture Condition	Extraction Kit	HRM Analysis	*k*3 (HRM)	PSQ results	*k*3 (rs16906252)	*k*4 (rs16906252)
GL16	brain, GBM	RPMI-1640, 7% FCS	QiaAmp	CCC/CCC	cluster a	n.a.	-	-
GL17	brain, GBM	RPMI-1640, 7% FCS	QiaAmp	CCC/CCC	cluster a	CCC/CCC	cluster 1	cluster 1
GL18	brain, GBM	RPMI-1640, 7% FCS	QiaAmp	CCC/CCC	cluster a	n.a.	-	-
GL19a	brain, GBM	RPMI-1640, 7% FCS	QiaAmp	CCC/CCC	cluster a	n.a.	-	-
GL19b	brain, rec. GBM	RPMI-1640, 7% FCS	QiaAmp	CCC/CCC	cluster a	CCC/CCC	cluster 1	cluster 1
GL19c	brain, rec. GBM	RPMI-1640, 7% FCS	QiaAmp	CCC/CCC	cluster a	n.a.	-	-
GL19d	brain, rec. GBM	RPMI-1640, 7% FCS	QiaAmp	CCC/CCC	cluster a	n.a.	-	-
GL19e	brain, rec. GS	RPMI-1640, 7% FCS	QiaAmp	CCC/CCC	cluster a	CCC/CCC	cluster 1	cluster 1
GL20a	brain, AA	RPMI-1640, 7% FCS	QiaAmp	CCC/CCC	cluster a	n.a.	-	-
GL20b	brain, sec. GBM	RPMI-1640, 7% FCS	QiaAmp	CCC/CCC	cluster a	n.a.	-	-
GL21	brain, DA	RPMI-1640, 7% FCS	QiaAmp	CCT/CCT	cluster b	CCT/CCT	cluster 3	cluster 3
GL22	brain, DA	RPMI-1640, 7% FCS	QiaAmp	CCC/CCC	cluster a	CCC/CCC	cluster 1	cluster 1
GL23a	brain, DA	RPMI-1640, 7% FCS	QiaAmp	CCC/CCC	cluster a	n.a.	-	-
GL23b	brain, OA	RPMI-1640, 7% FCS	QiaAmp	CCC/CCC	cluster a	CCC/CCC	cluster 1	cluster 1
GL24a	brain, GA	RPMI-1640, 7% FCS	QiaAmp	CCC/CCC	cluster a	CCC/CCC	cluster 1	cluster 1
GL24b	brain, AA	RPMI-1640, 7% FCS	QiaAmp	CCC/CCC	cluster a	CCC/CCC	cluster 1	cluster 1
GL25	brain, AA	RPMI-1640, 7% FCS	QiaAmp	CCC/CCC	cluster a	n.a.	-	-
GL26	brain, AA	RPMI-1640, 7% FCS	QiaAmp	CCC/CCC	cluster a	CCC/CCC	cluster 1	cluster 1

AA: anaplastic astrocytoma, DA: diffuse astrocytoma, FCC: fibrocystic changes, FCS: fetal calf serum, GA: gemistocytic astrocytoma, GBM: glioblastoma, GS: gliosarcoma,, HS horse serum, n.a.: not analyzed, OA: oligoastrocytoma, P/S: penicillin/streptomycin, rec.: reccurent, sec.: secondary, wt: wildtype.

Media were supplemented (final concentration) with FCS or HS, P/S or C: 0.1 µg/ml cholera toxin, E: 20 ng/µl EGF, G: 2 mM Gluatmin, H: 0.5 µg/ml hydrocortisone, I: 10 µg/ml insulin, N: 1x Non-essential Amino Acid Solution, P: 1 mM pyruvat. All media and supplements were purchased from Thermo Scientific (Vienna, Austria) or Sigma-Aldrich (Steinheim, Germany), except MEGM (Lonza, Basel, Swiss) and $NaHCO_3$ (VWR, Vienna, Austria).

† AU565 and SK-BR-3 derived from the same patient.

‡ HMEC derived from a patient undergoing reduction mammoplasty with no known breast pathology.

§ MCF 10A and MCF 10F are the adherent and floating sub cell line respectively.

¶ Medium 1 is a mixture of DMEM with10% NCTC medium and medium 2 is a 1:1 mixture of DMEM and Ham's F12.

Table S3. Genotyping results (3 SNPs assay) for breast cancer patients (BRC) and patients undergoing breast reduction mammoplasty (BRM).

Sample	HRM Analysis	*k*2 (HRM)	PSQ results	*k*3 (rs16906252)	*k*4 (rs16906252)
BRC01	CCC/CCC	cluster A	CCC/CCC	cluster 1	cluster 1
BRC02	CCC/CCC	cluster A	n.a.	-	-
BRC03	CCC/CCC	cluster A	n.a.	-	-
BRC04	CCC/CCC	cluster A	CCC/CCC	cluster 1	cluster 1
BRC05	CCC/CCC	cluster A	n.a.	-	-
BRC06	CCC/CCC	cluster A	n.a.	-	-
BRC07	CCC/CCC	cluster A	n.a.	-	-
BRC08	CCC/CCC	cluster A	n.a.	-	-
BRC09	CCC/CCT	cluster B	CCC/CCT	cluster 2	cluster 2
BRC10	CCC/CCC	cluster A	n.a.	-	-
BRC11	CCC/CCC	cluster A	CCC/CCC	cluster 1	cluster 1
BRC12	CCC/CCC	cluster A	CCC/CCC	cluster 1	cluster 1
BRC13	CCC/CCT	cluster B	CCC/CCT	cluster 2	cluster 2
BRC14	CCC/CCC	cluster A	CCC/CCC	cluster 1	cluster 1
BRC15	CCC/CCC	cluster A	n.a.	-	-
BRC16	CCC/CCC	cluster A	CCC/CCC	cluster 1	cluster 1
BRC17	CCC/CCT	cluster B	CCC/CCT	cluster 2	cluster 2
BRC18	CCC/CCC	cluster A	CCC/CCC	cluster 1	cluster 1
BRC19	CCC/CCC	cluster A	n.a.	-	-
BRC20	CCC/CCC	cluster A	CCC/CCC	cluster 1	cluster 1
BRC21	CCC/CCC	cluster A	n.a.	-	-
BRC22	CCC/CCC	cluster A	CCC/CCC	cluster 1	cluster 1
BRC23	CCC/CCC	cluster A	n.a.	-	-
BRC24	CCC/CCC	cluster A	n.a.	-	-
BRM01	CCC/CCC	cluster A	CCC/CCC	cluster 1	cluster 1
BRM02	CCC/CCC	cluster A	n.a.	-	-
BRM03	CCC/CCC	cluster A	n.a.	-	-
BRM04	CCC/CCT	cluster B	CCC/CCT	cluster 2	cluster 2
BRM05	CCC/CCC	cluster A	n.a.	-	-
BRM06	CCC/CCC	cluster A	n.a.	-	-
BRM07	CCC/CCC	cluster A	n.a.	-	-
BRM08	CCC/CCT	cluster B	CCC/CCT	cluster 2	cluster 2
BRM09	CCC/CCT	cluster B	CCC/CCT	cluster 2	cluster 2
BRM10	CCC/CCC	cluster A	CCC/CCC	cluster 1	cluster 1
BRM11	CCC/CCC	cluster A	n.a.	-	-
BRM12	CCC/CCC	cluster A	CCC/CCC	cluster 1	cluster 1
BRM13	CCC/CCT	cluster B	CCC/CCT	cluster 2	cluster 2
BRM14	CCC/CCC	cluster A	n.a.	-	-
BRM15	CCC/CCC	cluster A	n.a.	-	-
BRM16	CCC/CCC	cluster A	CCC/CCC	cluster 1	cluster 1
BRM17	CCC/CCC	cluster A	n.a.	-	-
BRM18	CCC/CCC	cluster A	n.a.	-	-
BRM19	CCC/CCT	cluster B	CCC/CCT	cluster 2	cluster 2
BRM20	CCC/CCC	cluster A	n.a.	-	-
BRM21	CCC/CCC	cluster A	CCC/CCC	cluster 1	cluster 1
BRM22	CCC/CCC	cluster A	CCC/CCC	cluster 1	cluster 1
BRM23	CCC/CCC	cluster A	CCC/CCC	cluster 1	cluster 1
BRM24	CCC/CCC	cluster A	CCC/CCC	cluster 1	cluster 1
BRM25	CCC/CCC	cluster A	CCC/CCC	cluster 1	cluster 1
BRM26	CCC/CCC	cluster A	CCC/CCC	cluster 1	cluster 1

Table S4. Genotyping results (3 SNPs assay) for samples from the NHGRI Sample Repository for Human Genetic Research.

Sample	ENSEMBL	HRM Analysis	*k*5 (HRM)	PSQ Results	*k*3 (rs527559815)	*k*3 (rs547832288)	*k*3 (rs16906252)	*k*4 (rs16906252)
HG00246	CCC/CCC	CCC/CCT	cluster γ	CCC/CCT	cluster 1	cluster 1	cluster 2	cluster 2
HG01241	CCC/CCC	CCC/CCC	cluster α	CCC/CCC	cluster 1	cluster 1	cluster 1	cluster 1
NA19095	CCC/TCC	CCC/TCC	cluster β	CCC/TCC	cluster 2	cluster 1	cluster 1	cluster 1
NA19175	CCC/TCC	CCC/TCC	cluster β	CCC/TCC	cluster 2	cluster 1	cluster 1	cluster 1
HG00239	CCC/CCT	CCC/CCT	cluster γ	CCC/CCT	cluster 1	cluster 1	cluster 2	cluster 2
HG00384	CCC/CCT	CCC/CCT	cluster γ	CCC/CCT	cluster 1	cluster 1	cluster 2	cluster 2
HG01784	CCT/CCT	CCT/CCT	cluster δ	CCT/CCT	cluster 1	cluster 1	cluster 3	cluster 3
NA19664	CCT/CCT	CCT/CCT	cluster δ	CCT/CCT	cluster 1	cluster 1	cluster 3	cluster 3
HG01605	CCC/CTT	CCC/CTT	cluster ε	CCC/CTT	cluster 1	cluster 2	cluster 2	cluster 2
